# Evaluating large language models for lay summaries of radiology reports using tailored prompting strategies and mixed-method assessment

**DOI:** 10.1371/journal.pdig.0001672

**Published:** 2026-09-17

**Authors:** Nanziba Tasneem, Christian B. van der Pol, Ambreen Zahoor, Nitin Juggath, Kyle McGowan, Cynthia Lokker, Ashirbani Saha

**Affiliations:** 1 MSc eHealth Program, McMaster University, Hamilton, Ontario, Canada; 2 Department of Medical Imaging, McMaster University, Hamilton, Ontario, Canada; 3 Department of Diagnostic Imaging, Juravinski Hospital and Cancer Centre, Hamilton Health Sciences, Hamilton, Ontario, Canada; 4 Escarpment Cancer Research Institute, Hamilton Health Sciences and McMaster University, Hamilton, Ontario, Canada; 5 Health Information Research Unit, Department of Health Research Methods, Evidence, and Impact, Faculty of Health Sciences, McMaster University, Hamilton, Ontario, Canada; 6 Department of Oncology, McMaster University, Juravinski Cancer Centre, Hamilton, Ontario, Canada; 7 CentRE for dAta science and digiTal hEalth (CREATE), Hamilton Health Sciences, Hamilton, Ontario, Canada; North Carolina A&T State University: North Carolina Agricultural and Technical State University, UNITED STATES OF AMERICA

## Abstract

Radiology reports are often filled with medical jargon that limits patient understanding. Lay summaries can improve understanding but are time-consuming for healthcare providers to create. The objective of this study is to explore the use of tailored prompts for five Large Language Models (LLMs) in generating lay summaries from radiology reports. Using 100 reports from the publicly available “BioNLP 2023 report summarization” dataset, lay summaries were generated by each LLM, under select prompting styles [*Few-Shot* (GPT-4), *Generated Knowledge* (GPT-4o mini, Gemini 1.5 – Pro, Gemini 1.5 – Flash), and *Zero-Shot* (Llama 3.1)] informed by a pilot work. The summaries were evaluated using a mixed-method framework: subjective assessment (Likert statements) by blinded experts (n = 2 radiology fellows) and Large Reasoning Models (LRMs) [(Gemini 2.5 – Pro (LRM 1); GPT-oss-120b (LRM 2)], and readability metrics (Flesch-Kincaid Grade Level and Flesch Reading Ease). Using percentage agreement of Likert statements, the LLM-prompt combinations’ performances were ranked, and Friedman and post-hoc Nemenyi tests were conducted. Gemini 1.5 - Flash and - Pro *(generated knowledge)* were rated highest by human experts and LRMs for generating actionable lay summaries that require minimal supervision [P < 4.97 × 10^-2^ (Rater 1); P < 9.03 × 10^-21^ (Rater 2), P < 6.90 × 10^-15^ (LRM 1), *P* < 2.760 × 10^-5^ (LRM 2). GPT-4 (*few-shot*) achieved the highest human-rated accuracy (98%), while Gemini 1.5 – Flash (LRM 1-rated: 95%) and Gemini 1.5 – Pro (LRM 2-rated: 91%) ranked first in LRM-rated accuracy. Gemini 1.5 - Pro produced the most accessible summaries (Flesch-Kincaid Grade Level: 7.55 ± 1.38, Flesch Reading Ease: 67.84 ± 7.78). Strong agreement was observed between experts and LRMs [0.96% (LRM 1) and 3.4% (LRM 2) complete disagreement]. Overall, this study highlights Gemini-models with *generated knowledge* prompts and the potential of LRM evaluators in assessing LLM-generated lay summaries.

## Author summary

Creating plain-language medical summaries for patients is important, but it adds extra work for healthcare providers. While previously Artificial Intelligence systems, Large Language Models (LLM), were used to create patient-friendly summaries, this study is the first to explore how tailored prompt styles affect the model performance in generating summaries from radiology reports. Five models were prompted using pilot study-informed tailored styles: 1. What the model “knows” (*Generated knowledge* prompt for Gemini 1.5 - Flash, Gemini 1.5 – Pro and GPT-4o mini), 2. Guided examples (*Few-shot* for GPT-4) and 3. Only instructions (*Zero-shot* for Llama 3.1), to generate summaries for patients. These summaries were evaluated by human experts (radiology fellows), Gemini 2.5 – Pro and GPT-oss-120b [Large Reasoning Models (LRM)] and reading metrics. The results show that Gemini 1.5 – Flash and Gemini 1.5 – Pro with *Generated knowledge* prompting produced 7^th^-9^th^ grade level summaries that had actionable and beneficial information and were more trusted by experts. We found that GPT-4 with *Few-shot* prompting produced the most accurate lay summaries according to the human raters. There was a strong similarity in ratings between the human experts and LRM evaluators. These findings show the potential of using AI to generate and evaluate clinical text summarization.

## Introduction

Patient imaging reports have a high degree of medical jargon, which is a common source of confusion among patients [1]. While the target audience for these reports is other providers, patients and their families also rely on them [2]. Inclusion of medical jargon can reduce patients’ understanding of their disease, affect their compliance with the treatment and increase the patient-provider communication gap [1]. Patient advocacy groups recommend including a patient-friendly summary in official medical documentation that is clear, easy to understand and uses simple language, i.e., a lay summary [3].

However, drafting and incorporating lay summaries would require additional staff capacity and attention, which most clinical fields cannot provide, particularly fields like radiology that are already facing increasing service demands [4]. In radiology workflows involving report development, pre-trained Large Language Models (LLMs), a revolutionary development in Artificial Intelligence (AI), have already been regarded as a cost-effective and powerful tool [5]. Using prompt engineering, a technique where users can design the request made to an LLM in order to generate the desired output, LLMs can be leveraged to generate lay summaries for imaging reports [6]. Different prompt engineering styles such as *zero-shot,* instructions with no examples, *few-shot,* instruction with some guided examples, and *generated knowledge*, typically, a two-step instruction where LLM generates useful information and then uses that information to perform the task, can also be explored for lay summary generation [7,8].

There are currently several studies that have conducted comparative analyses of LLMs’ performance in generating lay summaries from clinical text. Using the query “(Large Language Model) AND (Lay Summary) OR (Simplified) AND (Radiology),” research journals were searched. All studies, published between November 2023 and August 2025, mostly used a *zero-shot* prompting style to generate lay summaries from clinical text and evaluated the generated lay summaries using a mix of quantitative metrics (readability scores) and expert judgement (radiologists) [9–13]. One study used a *few-shot* prompting style on the GPT-3.5 model to generate lay summaries [10]. These studies also found GPT family models (GPT-3.5 or GPT-4) to be the best performing model at generating lay summaries of clinical reports. These studies focus on LLM-only performances, while a few emerging studies have focused on prompt-only performances such as creating training guidelines for clinicians on prompting styles or testing various prompts on a selected LLM on non-medical summarization tasks [14,15]. Given the known sensitivity of LLMs to prompt styles, there is a lack of investigation into the different LLM-prompt combinations for generating lay summaries, as they would be used in real-world practice, and the use of mixed–evaluation frameworks that align clinical expert judgement with the reasoning capabilities of Large Reasoning Models (LRMs) [16,17]. LRMs are a type of LLM that have been fine-tuned for advanced chain-of-thought reasoning and can generate internal reasoning steps along with the requested output [18]. Therefore, a systematic evaluation using LRMs on the performance of popular LLMs combined with tailored prompting styles, as real-world units, is required to inform clinical reporting workflows.

In this study, we performed a comparative analysis on LLM-prompt combinations in generating lay summaries for their inclusion of beneficial, supplementary and extra information, accuracy, conciseness, and readability. The summaries were evaluated subjectively by human raters (radiology fellows) and LRMs (Gemini 2.5 – Pro and GPT-oss-120B), and objectively through readability metrics. We addressed the literature gap by selecting a unique combination of LLMs, using various prompting styles meticulously selected for each LLM through a pilot work, and using LRM evaluators (in order to improve the readability of this paper and distinguish between the models, we will refer to the evaluator models as an “LRM” as they are the only models, used in our study, with reasoning generation capabilities). The aim of this study was to assess whether five LLMs, under the corresponding informed best tailored prompting strategy [GPT-4 (*few-shot*), GPT-4o mini, Gemini 1.5 – Pro, Gemini 1.5 – Flash (*generated knowledge*) and Llama 3.1 (*zero-shot*)], differ in performance at generating lay summaries for radiology imaging reports based on evaluations by humans, LRMs, and quantitative tests. Our results demonstrate that this aim was successfully achieved.

## Materials and methods

### Ethics statement

The Hamilton Integrated Research Ethics Board (#18001) approved this study. We used the “BioNLP 2023 report summarization” dataset sourced from MIMIC Chest X-ray Database v2.0.0 [19]. The dataset was used to *only* prompt LLMs that explicitly state that they do not store or use consumer data to train or fine-tune the models [20–22]. This complies with PhysioNet’s condition that the dataset would not be used to train any models as an exception to the GPT-Responsible Use Policy [23].

### Dataset preparation

A convenience sample of 100 reports was made using the training set of the dataset, which contains 65,846 radiology report samples with findings and impressions (Training Set: 59,320; Test Set: 6,526). The sample was created by first stratifying radiology reports in the Training Set into five categories (0–400, 400–800, 800–1200, 1200–2000, and 2000–8000 characters) based on the character count of the “findings” and then randomly sampling 20 reports from each category.

### Selection of Large Language and Reasoning Models

The study used five LLMs (GPT-4, GPT-4o mini, Gemini 1.5 – Pro, Gemini 1.5 – Flash, and Llama 3.1) and two LRMs (Gemini 2.5 – Pro and GPT-oss-120B). The LLMs were selected based on the results of a pilot study (Table A in S1 Text), recent release dates (2024) and availability of Application Programming Interface (API). The 2023 release of GPT-4 was included given its demonstrated success in existing literature in generating lay summaries. For each LLM and its tailored prompt, we utilized the most current version available during lay summary generation (November 17–20, 2024). Additionally, the LRMs were selected because of their strong performance in reasoning benchmarks, Humanity’s Last Exam (Gemini 2.5 – Pro: 21.6%; GPT-oss-120B: 18.5%) and Measuring Massive Multitask Language Understanding (Gemini 2.5 – Pro: 84.1%; GPT-oss-120B: 81.3%), at the time of evaluation (Gemini 2.5 – Pro: June 18, 2025; GPT-oss-120B: February 7–15, 2026) [24–27].

### Lay summary generation

**Fig 1:**
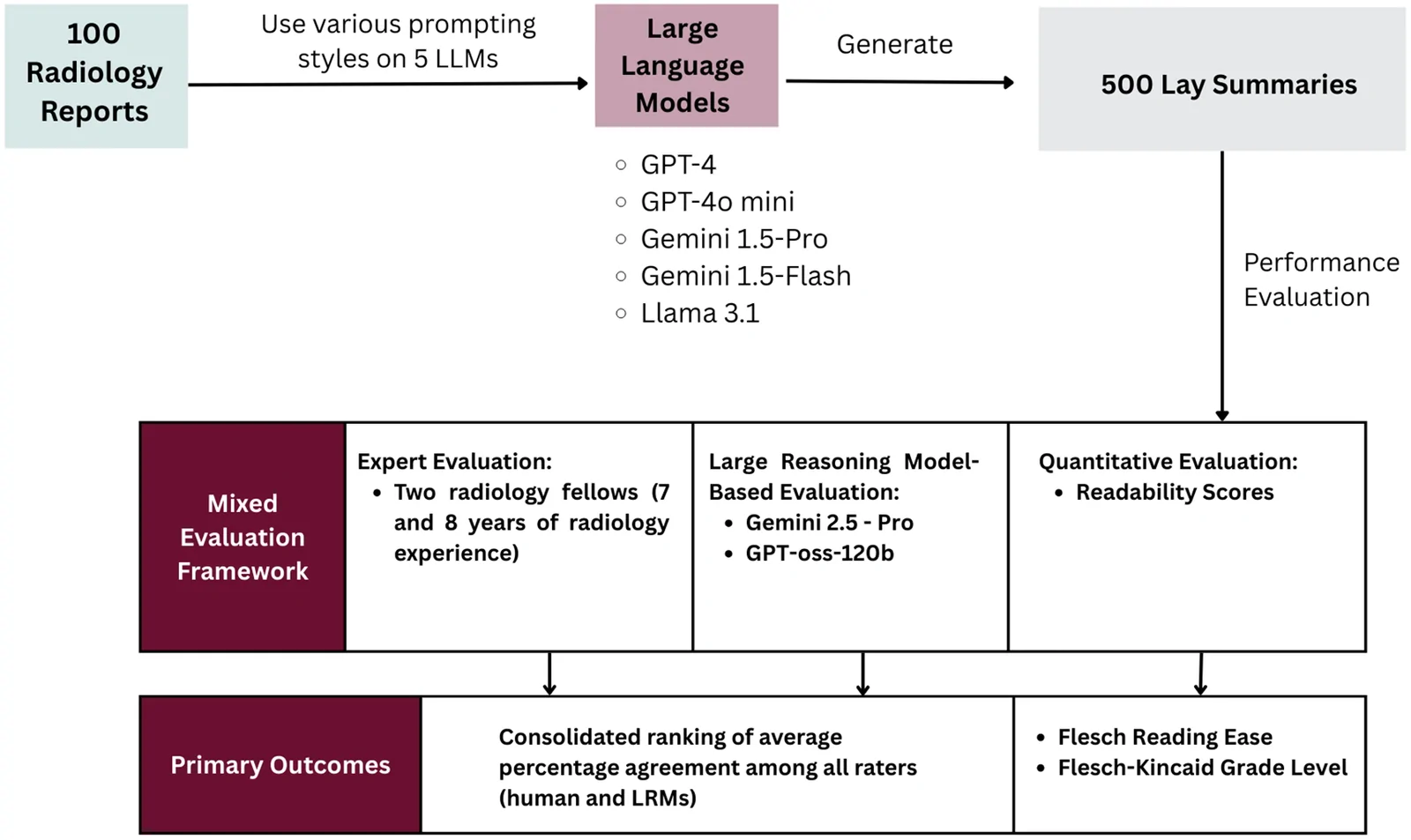
Methodology (process map) of the generation of lay summaries by Large Language Models (LLMs) and evaluation by human experts and Large Reasoning Models (LRMs).

All LLMs were prompted using Python scripts and API calls (Fig 1). The models were instructed via one of the three prompt engineering styles (*zero-shot, few-shot* and *generated knowledge*) to generate lay summaries for patients using “findings” and “impression” sections of the radiology reports. The most appropriate (of the three) prompt engineering styles and hyperparameter combinations [Temperature and Top_p (0.25, 0.5, 0.75 or 1.0)] per model were determined through prior pilot work that was also used in a previously published paper (S2 Text) [28]. The prompts and hyperparameters of each model have been summarized in Table 1.

**Table 1:** Large Language Models (LLMs) and their hyperparameters, prompt engineering styles, and prompts.

LLMs (Hyperparameters)	Prompt Engineering Styles	Prompt
Llama 3.1 (Temperature: 0.5; Top_p: 1.0)	Zero-shot	Act as an educator and write a lay summary using the findings and impressions. The goal is to simplify the radiology report for non-experts and make the report easily accessible and understandable for the broader public, patient and patient’s family. The summary should be written in plain English so that it understandable at different reading levels. Avoid jargon and acronyms. Explain any complex terminology if important to the lay summary. Create simple sentences without many clauses. Use an active voice. Use clear and concise language. Avoid ambiguity. Write the lay summary in a logical manner. Organize the lay summary in sections where appropriate.
GPT-4 (Temperature: 0.5; Top_p: 1.0)	Few-shot	Act as an educator and write a lay summary using the findings and impression. The goal is to simplify the radiology report for non-experts and make the report easily accessible and understandable for the broader public, patient and patient’s family. The summary should be written in plain English so that it understandable at different reading levels. Avoid jargon and acronyms. Explain any complex terminology if important to the lay summary. Create simple sentences without many clauses. Use an active voice. Use clear and concise language. Avoid ambiguity. Write the lay summary in a logical manner. Organize lay summary in sections where appropriate. Use these examples as a guide: < examples>
**•** Gemini 1.5 – Flash (Temperature: 0.75; Top_p: 1.0) **•** Gemini 1.5 – Pro (Temperature: 0.75; Top_p: 0.75) **•** GPT-4o mini (Temperature: 1.0; Top_p: 0.75)	Generated Knowledge (Single-step generation)	Act as an educator and write a concise lay summary using the findings and impression. The goal is to simplify the radiology report for non-experts and make the report easily accessible and understandable for the broader public, patient and patient’s family. Consider what a good lay summary should have. Apply these considerations into generating a lay summary.

These prompts were developed using literature and guidelines for lay summaries of clinical reports [29,30]. Here, the *generated knowledge* prompt is a single step generation [30] given the subjectivity involved in the process of summarization where the models’ generated knowledge is not explicitly printed but applied. The responses from the LLMs were trimmed for any conversational fillers, ensuring that only the lay summary would be available for evaluation.

### Performance evaluation

The LLM-generated lay summaries were evaluated using a mixed evaluation framework of three modes (Fig 1):

(a) Human expert evaluation of lay summaries: Using REDCap [31,32] electronic data capture tools at McMaster University, a total of 500 lay summaries were evaluated by each of two radiology fellows with 7 and 8 years of radiology experience. The human raters were blinded to the identity of the models. The survey questions (S3 Text) were developed using published literature and feedback from a radiologist team member who previously evaluated 30 LLM-generated lay summaries (from non-overlapping reports in the test set of the dataset) [9,29]. The assessment included a 5-point Likert scale survey (1 = Strongly Disagree, 2 = Disagree, 3 = Neither Agree or Disagree, 4 = Agree, 5 = Strongly Agree) on five primary statements (criteria) [9,29]:
  1 The simplified version does not contain any inaccurate or misleading information. (This statement assesses the Inaccurate Information criterion.)
  2 The simplified version includes all relevant/actionable information present in the original impression. (This statement assesses the Actionable Information criterion.)
  3 The simplified version offers beneficial supplementary information not found in the original impression. (This statement assesses the Beneficial Supplementary Information criterion.)
  4 The simplified version of the radiology report contains extraneous information. (This statement assesses the Extraneous Information criterion.)
  5 I feel comfortable giving the simplified version to patients without any supervision. (This statement assesses the No Supervision criterion.)

The Likert scale option “3 = Neither Agree or Disagree” is referred to as “Neutral” throughout the document in order to improve visualization in figures and ease of discussion in the results.

Additionally, the human experts assessed whether the generated lay summaries were accurate and concise. These criteria measured whether the LLM generated lay summaries correctly and faithfully shortened the key clinical content of the source text, without omitting clinically relevant information or hallucinating details. The experts also answered three overall questions comparing the five LLM-generated lay summaries per radiology report. The questions include: “Which summary completely captures more important information?”, “Which summary includes less false information?”,and “Which summary contains less non-important information?” [29]

(b) LRM evaluation of lay summaries: All 500 lay summaries were evaluated using Gemini 2.5 – Pro (LRM 1) and GPT-oss-120B (LRM 2) [33,34]. Two LRMs from different model families and companies, OpenAI and Google, were used to avoid instances of model self-preference bias and to strengthen consistency in the evaluation [35]. The LRMs were blinded to the identities of the LLMs and provided the original radiology report for context. Gemini 2.5 – Pro was accessed using the Vertex AI platform while GPT-oss-120B was downloaded locally. A role-based prompt with the same evaluation criteria of questions and Likert statements given to the human raters (S3 Text) was used. The hyperparameter settings (Temperature and Top_p) were kept at the default option [33,34].
(c) Quantitative evaluation of lay summaries: Readability of the generated lay summaries and the original report was calculated using a variety of measures. These measures signify different characteristics of the text (sentence length vs syllable count, etc.) therefore, using a variety can provide a complete understanding of text readability [36]. The readability measures calculated are as follows:
  - Flesch Reading Ease (FRE) - calculated using the average sentence length and average syllables for sentence complexity and ranges between 0 and 100. Higher scores indicate easier reading [37].
  - Flesch-Kincaid Grade Level (FKGL) – calculated using similar components as FRE and used to indicate the United States school grade-level required to understand text [34].
  - Simple Measure of Gobbledygook (SMOG) index – calculated using polysyllabic words in sentences to determine grade level [38].
  - Automated Readability Index (ARI) - calculated using characters per word and words per sentence to determine U.S grade level [38].
  - Dale-Chall Readability Score – calculates score based on 3000 common words. Lower scores indicates easier readability [38].

The primary outcomes of this study include the consolidated ranking of average percentage agreement among all raters (human and LRM raters together), and the readability metrics (FRE and FKGL).

### Statistical analysis

The LLM-specific median and Interquartile Range (IQR) of the Likert criteria were determined for the first and second modes of evaluation. Statistical tests (Friedman test and post-hoc Nemenyi test) were conducted to assess the significance of each rater’s ratings for each pair of models on a criterion. The percentage agreeability of agree and strongly agree responses to Likert statements was calculated per LLM and criterion based on the raters’ responses. Frequency counts for accuracy, conciseness, and overall comparison of the evaluations were conducted. To determine each raters’ agreement with other raters, confusion matrices between the human raters and the LRMs were calculated. Inter-rater agreement metrics, intraclass correlation coefficients - single fixed raters (ICC3) and average fixed raters (ICC3k) were calculated between the human raters. For associations between the character length of the “findings” section, i.e., prompt length and the quality of the generated lay summaries, Spearman’s rank correlation for the Likert statements responses and a Mann-Whitney U test for the accuracy responses were conducted.

For the third mode of evaluation, the average of readability metrics and character count were calculated for the original report and lay summaries. To determine significant variations in performance, a Friedman test and the post-hoc Nemenyi test were conducted on the readability scores. The level of significance for all statistical tests was set at 0.05.

## Results

### First mode (Human experts’ {radiology fellows} evaluation of generated lay summaries)

**Fig 2:**
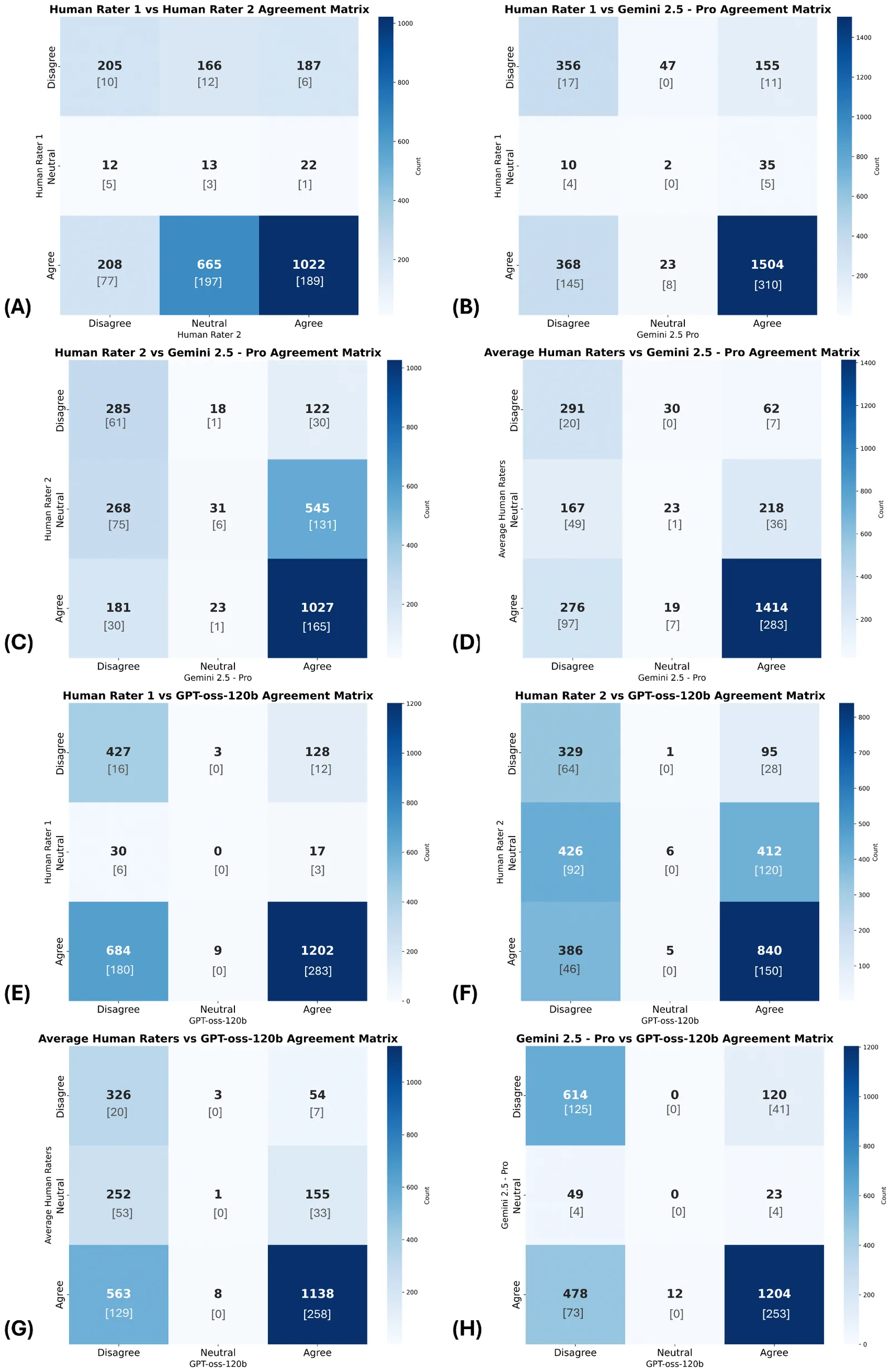
Count of overall agreeability (in bold) and No Supervision criterion [“I feel comfortable giving the simplified version to patients without any supervision” (in [] brackets)] ratings between a pair of raters (either one of the human raters, average human ratings, or a large reasoning model) for evaluating lay summaries generated by large language models. Agreement matrices showing count comparisons between **(A)** Human Raters 1 and 2, **(B)** Rater 1 and Gemini 2.5 - Pro, **(C)** Rater 2 and Gemini 2.5 - Pro, **(D)** averaged human ratings and Gemini 2.5 - Pro **(E)** Rater 1 and GPT-oss-120b, **(F)** Rater 2 and GPT-oss-120b, **(G)** averaged human ratings and GPT-oss-120b, and **(H)** Gemini 2.5 -Pro and GPT-oss-120b.

Based on Table 2, both human raters (Rater 1 and Rater 2) rated lay summaries by Gemini-1.5 Flash and Gemini 1.5 – Pro (*generated knowledge prompts*) with high performing scores (4.00 or 5.00 median scores for all criteria except “Extraneous Information,” where 1.00 or 2.00 median scores indicate better performance) compared to other LLM-prompt combinations (S1 Data). Additionally, using a consolidated ranking of the percentage agreement between the human raters on the five Likert criteria, Gemini-1.5 Flash (*generated knowledge)* ranked first, followed by Gemini 1.5 – Pro (*generated* *knowledge)*, GPT-4o mini (*generated knowledge*) and GPT-4 (*few-shot*). Gemini 1.5 – Flash and Gemini 1.5 – Pro with *generated knowledge* prompts ranked consistently first and second, respectively, among all criteria except for the “Extraneous Information” criterion (Table A in S5 Text). More importantly, for the “No Supervision” criterion, Gemini 1.5 – Pro (*generated knowledge)* had the highest percentage agreeability (71%), followed closely by Gemini 1.5 – Flash (*generated knowledge;* 70%) compared to the other models (<30%) (Table 2). GPT-4o mini (*generated knowledge)* and GPT-4 (*few-shot*) ranked 3^rd^ and 4^th^ interchangeably among the criteria while Llama 3.1 (*zero-shot*) consistently ranked last in all criteria. There was strong agreement overlap between human raters, though Rater 2 relied more often on the Neutral option than Rater 1 (Fig 2). In total, there were 213 instances (8.5%) of polarizing opinions (Agree vs. Disagree) on Likert statements between the two raters out of 2500 responses with mostly fair inter-rater agreement (< 0.529) (Table 3). Most disagreements between the raters were regarding Llama 3.1 – *zero-shot* prompted lay summaries (S1 Fig).

**Table 2:** Comparison of Likert scores across raters, models, and evaluation criteria with inter-rater agreement for large language model-generated lay summaries*.

Model (Tailored Prompt) and Evaluation Criteria	Human Rater 1 (Median [IQR])	Human Rater 2 (Median [IQR])	Gemini 2.5 – Pro: LRM Evaluator (Median [IQR])	GPT-oss-120b: LRM Evaluator (Median [IQR])	Percentage of time both human raters displayed agreeability to the criterion (Agreed or Strongly Agreed) (%)	Percentage of time raters (human and Gemini 2.5 - Pro) displayed agreeability to the criterion (Agreed or Strongly Agreed) (%) [Percentage Difference (Human Rater Only Agreeability - Human and LRM Agreeability)]	Percentage of time raters (human and GPT-oss-120b) displayed agreeability to the criterion (Agreed or Strongly Agreed) (%) [Percentage Difference (Human Rater Only Agreeability - Human and LRM Agreeability)]	Percentage of time both LRMs displayed agreeability to the criterion (Agreed or Strongly Agreed) (%)
Gemini 1.5 – Flash (*Generated Knowledge*)								
The simplified version does not contain any inaccurate or misleading information. (↑)	5.00 [5.00-5.00]	4.00 [4.00-4.00]	5.00 [5.00-5.00]	5.00 [4.00-5.00]	84%	82% [+2]	69% [+15]	77%
The simplified version includes all relevant/actionable information present in the original impression. (↑)	5.00 [5.00-5.00]	4.00 [4.00-4.00]	5.00 [5.00-5.00]	5.00 [4.00-5.00]	81%	76% [+5]	67% [+14]	78%
The simplified version offers beneficial supplementary information not found in the original impression. (↑)	4.00 [4.00-4.00]	4.00 [3.00-4.00]	5.00 [4.00-5.00]	2.00 [2.00-4.00]	57%	57% [0]	27% [+30]	41%
The simplified version of the radiology report contains extraneous information. (↓)	1.00 [1.00 – 1.00]	2.00 [2.00-3.00]	1.00 [1.00 – 2.00]	2.00 [1.00 – 2.00]	0%	0% [0]	0% [0]	0%
I feel comfortable giving the simplified version to patients without any supervision. (↑)	5.00 [5.00 – 5.00]	4.00 [3.00 – 4.00]	5.00 [5.00 – 5.00]	4.00 [2.00 – 5.00]	70%	60% [+10]	53% [+17]	63%
Gemini 1.5 – Pro (*Generated Knowledge*)								
The simplified version does not contain any inaccurate or misleading information. (↑)	5.00 [5.00-5.00]	4.00 [4.00-4.00]	5.00 [5.00-5.00]	5.00 [5.00-5.00]	80%	75% [+5]	71% [+9]	83%
The simplified version includes all relevant/actionable information present in the original impression. (↑)	5.00 [5.00-5.00]	4.00 [4.00-5.00]	5.00 [5.00-5.00]	5.00 [4.00-5.00]	78%	69% [+9]	61% [+17]	73%
The simplified version offers beneficial supplementary information not found in the original impression. (↑)	4.00 [3.00-5.00]	4.00 [3.00-4.00]	5.00 [4.00-5.00]	2.00 [2.00-4.00]	45%	45% [0]	18% [+27]	40%
The simplified version of the radiology report contains extraneous information. (↓)	1.00 [1.00 – 1.00]	2.00 [2.00-3.00]	1.00 [1.00 – 2.00]	2.00 [1.00 – 2.00]	0%	0% [0]	0% [0]	0%
I feel comfortable giving the simplified version to patients without any supervision. (↑)	5.00 [4.00 – 5.00]	3.75 [4.00 – 4.00]	5.00 [4.00 – 5.00]	4.00 [4.00 – 5.00]	71%	61% [+10]	54% [+17]	68%
GPT-4o mini (*Generated Knowledge*)								
The simplified version does not contain any inaccurate or misleading information. (↑)	5.00 [5.00-5.00]	4.00 [3.00-4.00]	5.00 [5.00-5.00]	5.00 [5.00-5.00]	67%	58% [+9]	59% [+8]	82%
The simplified version includes all relevant/actionable information present in the original impression. (↑)	5.00 [5.00-5.00]	4.00 [3.00-4.00]	5.00 [5.00-5.00]	5.00 [2.00-5.00]	62%	56% [+6]	46% [+16]	71%
The simplified version offers beneficial supplementary information not found in the original impression. (↑)	4.00 [4.00-5.00]	3.00 [3.00-4.00]	5.00 [4.00-5.00]	3.00 [2.00-4.00]	43%	43% [0]	20% [+23]	46%
The simplified version of the radiology report contains extraneous information. (↓)	1.00 [1.00 – 2.00]	3.00 [3.00-4.00]	2.00 [1.00 – 2.00]	2.00 [1.75 – 2.00]	1%	0% [+1]	0% [+1]	0%
I feel comfortable giving the simplified version to patients without any supervision. (↑)	5.00 [5.00 – 5.00]	3.00 [3.00 – 4.00]	5.00 [4.00 – 5.00]	4.00 [2.00 – 5.00]	29%	24% [+5]	21% [+8]	64%
GPT-4 (*Few-Shot*)								
The simplified version does not contain any inaccurate or misleading information. (↑)	5.00 [5.00-5.00]	4.00 [3.00-4.00]	5.00 [5.00-5.00]	5.00 [4.00-5.00]	66%	57% [+9]	54% [+12]	76%
The simplified version includes all relevant/actionable information present in the original impression. (↑)	5.00 [5.00-5.00]	4.00 [3.00-4.00]	5.00 [5.00-5.00]	5.00 [4.00-5.00]	65%	57% [+8]	55% [+10]	78%
The simplified version offers beneficial supplementary information not found in the original impression. (↑)	4.00 [4.00-4.00]	3.00 [3.00-4.00]	5.00 [5.00-5.00]	4.00 [2.00-5.00]	31%	30% [+1]	23% [+8]	61%
The simplified version of the radiology report contains extraneous information. (↓)	2.00 [1.00 – 2.00]	4.00 [3.00-4.00]	2.00 [1.00 – 4.00]	2.00 [2.00 – 2.00]	0%	0% [0]	0% [0]	8%
I feel comfortable giving the simplified version to patients without any supervision. (↑)	5.00 [4.00 – 5.00]	3.00 [3.00 – 3.00]	5.00 [2.00 – 5.00]	4.00 [2.00 – 5.00]	18%	13% [+5]	15% [+3]	52%
Llama 3.1 (*Zero-Shot*)								
The simplified version does not contain any inaccurate or misleading information. (↑)	5.00 [4.00-5.00]	3.00 [2.00-3.00]	2.00 [1.00-4.00]	1.00 [1.00-2.00]	16%	8% [+8]	4% [+12]	10%
The simplified version includes all relevant/actionable information present in the original impression. (↑)	4.00 [5.00-5.00]	3.00 [2.00-3.00]	2.00 [1.00-5.00]	2.00 [2.00-4.00]	21%	10% [+11]	8% [+13]	32%
The simplified version offers beneficial supplementary information not found in the original impression. (↑)	4.00 [4.00-5.00]	3.00 [2.00-3.00]	4.00 [4.00-5.00]	2.00 [2.00-4.00]	9%	9% [0]	4% [+5]	30%
The simplified version of the radiology report contains extraneous information. (↓)	2.00 [2.00 – 4.00]	4.00 [4.00-4.00]	4.00 [3.00-5.00]	4.00 [4.00-5.00]	27%	21% [+6]	24% [+3]	65%
I feel comfortable giving the simplified version to patients without any supervision. (↑)	4.50 [4.00 – 5.00]	2.00 [2.00 – 3.00]	1.00 [1.00 – 2.00]	2.00 [1.00 – 2.00]	1%	1% [0]	0% [+1]	6%

*Note: This table summarizes lay summary evaluation criteria with median and IQR Likert scores (1 = Strongly Disagree, 2 = Disagree, 3 = Neutral, 4 = Agree, 5 = Strongly Agree). Performance direction is indicated by arrows: (↑) higher Likert scores reflect superior performance; (↓) lower Likert scores reflect superior performance. Agreement percentages and score difference between human experts and the LRM evaluators are included.

**Table 3:** Inter-rater agreement metric using intraclass correlation coefficient - single fixed raters (ICC3) and average fixed raters (ICC3k) displayed using ICC (P-value) using 5-point scale.

Model (Tailored Prompt) and Evaluation Criteria	ICC3	ICC3K
Gemini 1.5 – Flash (*Generated Knowledge*)		
The simplified version does not contain any inaccurate or misleading information.	0.184 (0.03)	0.310 (0.03)
The simplified version includes all relevant/actionable information present in the original impression.	0.360 (0.00)	0.529 (0.00)
The simplified version offers beneficial supplementary information not found in the original impression.	0.188 (0.03)	0.317 (0.03)
The simplified version of the radiology report contains extraneous information.	0.121 (0.11)	0.216 (0.11)
I feel comfortable giving the simplified version to patients without any supervision.	0.283 (0.00)	0.441 (0.00)
Gemini 1.5 – Pro (*Generated Knowledge*)		
The simplified version does not contain any inaccurate or misleading information.	-0.059 (0.72)	-0.125 (0.72)
The simplified version includes all relevant/actionable information present in the original impression.	0.046 (0.32)	0.088 (0.32)
The simplified version offers beneficial supplementary information not found in the original impression.	0.175 (0.04)	0.298 (0.04)
The simplified version of the radiology report contains extraneous information.	-0.167 (0.95)	-0.401 (0.95)
I feel comfortable giving the simplified version to patients without any supervision.	0.080 (0.21)	0.149 (0.21)
GPT-4o mini (*Generated Knowledge*)		
The simplified version does not contain any inaccurate or misleading information.	0.086 (0.20)	0.159 (0.20)
The simplified version includes all relevant/actionable information present in the original impression.	0.129 (0.10)	0.229 (0.10)
The simplified version offers beneficial supplementary information not found in the original impression.	0.128 (0.10)	0.226 (0.10)
The simplified version of the radiology report contains extraneous information.	0.023 (0.41)	0.045 (0.41)
I feel comfortable giving the simplified version to patients without any supervision.	0.125 (0.11)	0.222 (0.11)
GPT-4 (*Few-Shot*)		
The simplified version does not contain any inaccurate or misleading information.	-0.124 (0.89)	-0.284 (0.89)
The simplified version includes all relevant/actionable information present in the original impression.	-0.075 (0.77)	-0.162 (0.77)
The simplified version offers beneficial supplementary information not found in the original impression.	0.000 (0.50)	0.000 (0.50)
The simplified version of the radiology report contains extraneous information.	0.153 (0.06)	0.266 (0.06)
I feel comfortable giving the simplified version to patients without any supervision.	-0.069 (0.75)	-0.148 (0.75)
Llama 3.1 (*Zero-Shot*)		
The simplified version does not contain any inaccurate or misleading information.	0.174 (0.04)	0.296 (0.04)
The simplified version includes all relevant/actionable information present in the original impression.	0.215 (0.02)	0.355 (0.02)
The simplified version offers beneficial supplementary information not found in the original impression.	0.146 (0.07)	0.256 (0.07)
The simplified version of the radiology report contains extraneous information.	0.111 (0.14)	0.199 (0.14)
I feel comfortable giving the simplified version to patients without any supervision.	0.113 (0.13)	0.203 (0.13)

Friedman test found that Likert scores of each criterion were significantly different among the models per rater [*P* < 4.97 × 10^-2^ (Rater 1); *P* < 9.03 × 10^-21^ (Rater 2)]. In the post-hoc Nemenyi test, Llama 3.1 *(zero-shot)* ratings significantly varied (*P* < 0.001) compared to other LLM-prompt combinations in the “Extraneous Information” criteria, for Rater 1. For Rater 2, significantly different model pairs were found for all criteria (*P* < 0.0001).

**Fig 3:**
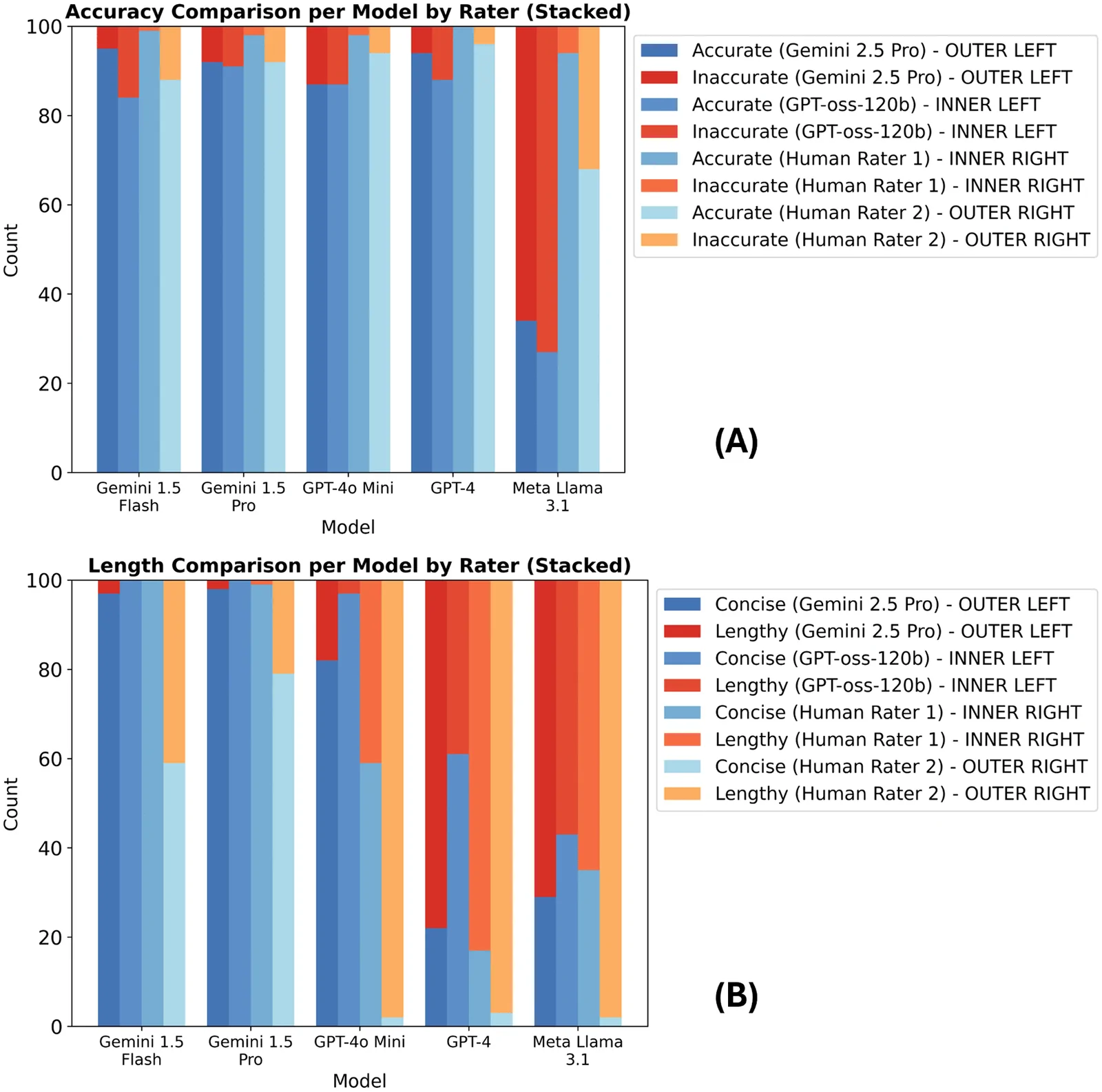
Comparison of accuracy and conciseness ratings (human raters and large reasoning model-based) of large language model-generated lay summaries. **(A)** Stacked bar plot of accuracy (accurate vs. inaccurate). **(B)** Stacked bar plot of length (concise vs. lengthy).

The raters were also asked to evaluate the accuracy and conciseness of the generated lay summaries. Based on the average of “Accurate” responses between both raters (Fig 3), GPT-4 *(few-shot)* generated lay summaries had the highest accuracy, followed by GPT-4o mini *(generated knowledge)*, Gemini 1.5 Pro (*generated knowledge)*, Gemini 1.5 Flash *(generated knowledge)* and Llama 3.1 *(zero-shot)* (98%, 96%, 95%, 93.5%, 81%). In the subjective evaluation of the conciseness of the lay summaries, Gemini 1.5 – Pro and Gemini 1.5 – Flash with *generated knowledge* prompts produced more concise lay summaries (89% and 79.5% average count between both raters). In comparison, GPT-4 with *few-shot* produced the lengthiest lay summaries (90% average count).

**Fig 4:**
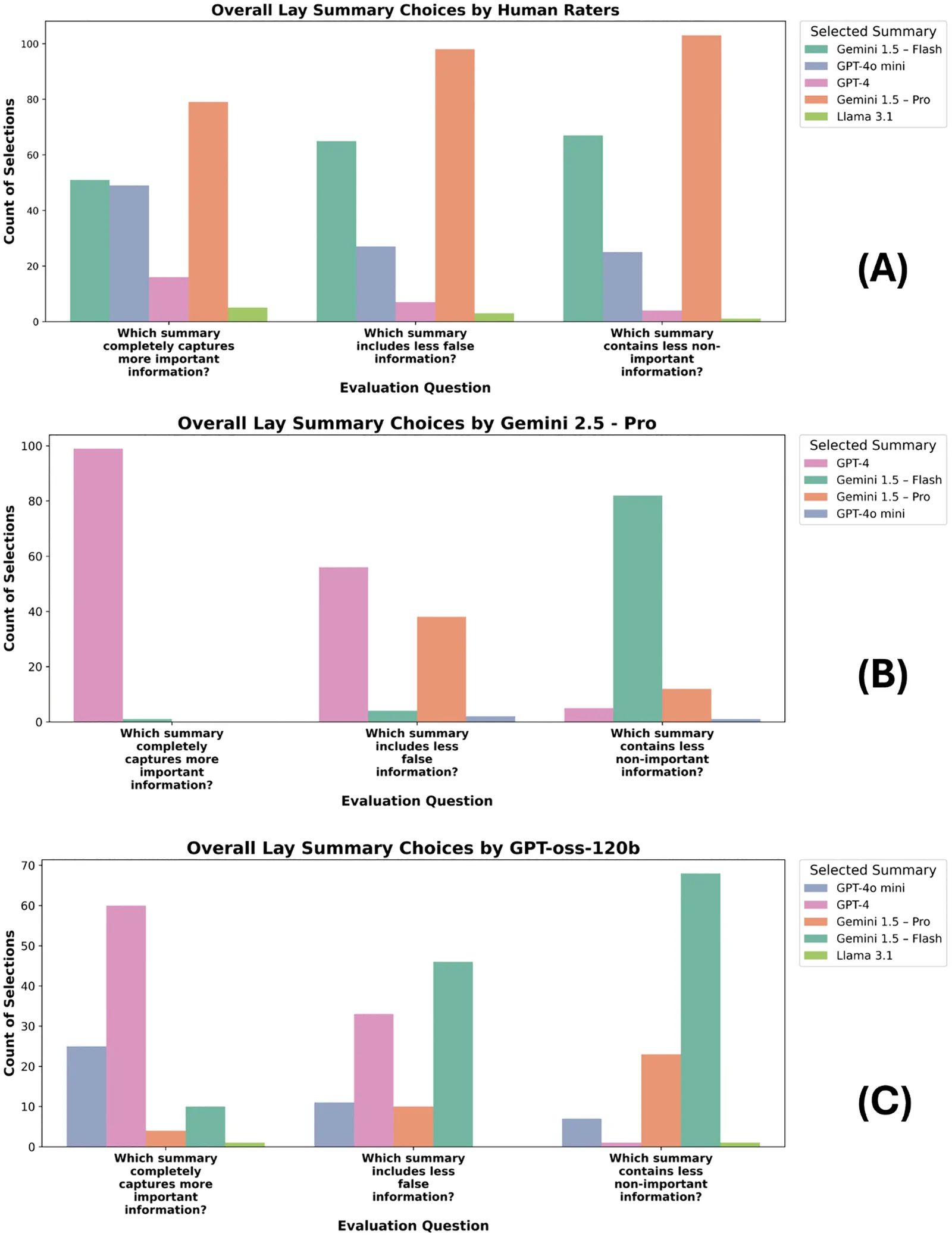
Count of choices per rater comparing Large Language Model (LLM)-generated lay summaries for 5 LLMs. **(A)** Depicts the combined number votes from both human raters. **(B)** Depicts the number of votes per question according to Gemini 2.5 – Pro. **(C)** Depicts the number of votes per question according to GPT-oss-120b.

In the overall comparison questions, Rater 1 found Gemini 1.5 – Flash (*generated knowledge)* summaries contained less non-important information (48) and false information (49), and GPT-4o mini summaries (*generated knowledge)* contained more important information (34). Comparatively, Rater 2 found that Gemini 1.5- Pro with *generated knowledge* prompt contained less non-important information (77) and false information (71) and more important information (55). Combining the votes of each rater across these questions (Fig 4), reveals that Gemini 1.5 – Pro, followed by Gemini 1.5 – Flash, both evaluated under the *generated knowledge* prompting strategy, ranked higher than the other models in overall quality.

### Second mode (LRM’s {Gemini 2.5 – Pro and GPT-oss-120b} evaluation of generated lay summaries)

From Table 2, Gemini 2.5 – Pro (LRM 1) rated all LLM-generated lay summaries with high performing scores for all Likert criteria except for Llama 3.1 with *zero-shot* (5.00 median scores for all criteria except the “Extraneous Information” criterion, where 1.00 or 2.00 median scores indicate better performance) (S1 Data). GPT-oss-120b (LRM 2) had more varied ratings than LRM 1 where it rated most LLM-generated lay summaries with generally high median scores (5.00) for “Inaccurate Information” and “Actionable Information” criteria (except Llama 3.1), and assigned lower median scores on the “Beneficial Supplementary Information” criterion for Gemini 1.5 – Flash and Pro with *generated knowledge* (2.00) (S1 Data). The Friedman test found that LRM 1 and LRM 2 ratings for all criteria significantly varied (LRM 1: *P* < 6.90 × 10^-15^; LRM 2: *P* < 2.760 × 10^-5^). The post-hoc Nemenyi test found that for both LRM raters, Llama 3.1 with *zero-shot* was significantly worse than others in all criteria (LRM 1: *P* < 3.20 × 10^-2^; LRM 2: *P* < 2.10 × 10^-5^) but LRM 1 found it to be significantly better at providing supplementary information (*P* < 0.0001).

Based on the consolidated average percentage agreeability ranking between the LRM raters and human raters, under their corresponding tailored prompting styles Gemini 1.5 - Flash *(generated knowledge)* ranked first, followed closely by Gemini 1.5 – Pro *(generated knowledge)*, and GPT-4o mini *(generated knowledge)* and GPT-4 *(few-shot)* ranked third equally while Llama 3.1 *(zero-shot)* ranked last (Tables B and C in S5 Text). Similar to the human expert ranking, with *generated knowledge prompts,* Gemini 1.5 – Pro had a higher percentage of agreeability (LRM 1: 61%; LRM 2: 54%) for the “No Supervision” criteria, followed closely by Gemini 1.5 – Flash (LRM 1: 60%; LRM 2: 53%) compared to the other models (<25%).

In Fig 2, the LRM 1 displayed strong agreement with Rater 1 (Agree-Agree: 1504; Disagree-Disagree: 356; Neutral-Neutral: 2), Rater 2 (Agree-Agree: 1027; Disagree-Disagree: 285; Neutral-Neutral: 31) and with the combined average rating of both human raters (Agree-Agree: 1414; Disagree-Disagree: 291; Neutral-Neutral: 23). The LRM 2 displayed strong agreement with Rater 1 (Agree-Agree: 1202; Disagree-Disagree: 427; Neutral-Neutral: 0), Rater 2 (Agree-Agree: 840; Disagree-Disagree: 329; Neutral-Neutral: 6) and with the combined average rating of both human raters (Agree-Agree: 1138; Disagree-Disagree: 326; Neutral-Neutral: 1). LRM 1 and 2 also had a strong agreement (Agree-Agree: 1204; Disagree-Disagree: 614; Neutral-Neutral: 0). For the “No Supervision” criteria, while the LRMs largely “disagreed” with sharing the lay summaries without supervision where the human raters “agreed”, there were some instances where the LRMs agreed while the human raters disagreed (LRM 1-Rater 1: 11 summaries; LRM 1-Rater 2: 30 summaries; LRM 2-Rater 1: 12 summaries; LRM 2-Rater 2: 28 summaries). Overall, there were 0.96% (24/2500 instances) (LRM 1) and 3.4% (86/2500 instances) (LRM 2) polarizing disagreements where the LRMs disagreed with both raters, while there were 25% (632/2500 instances) (LRM 1) and 36% (921/2500 instances) (LRM 2) polarizing disagreements where the LRMs disagreed with at least one rater

For the criterion of accuracy, LRM 1 found Gemini 1.5- Flash *(generated knowledge)* generated lay summaries to be most accurate (95%), followed by GPT-4 (*few-shot;* 94%) and Gemini 1.5 – Pro (*generated knowledge;* 92%) while the LRM 2 found Gemini 1.5 – Pro *(generated knowledge)* generated lay summaries to be most accurate (91%), followed by GPT-4 (*few-shot;* 88%) and GPT-4o mini (*generated knowledge;* 87%). (Fig 3). In comparison to human raters, there were some instances where the LRMs disagreed on the accuracy of the lay summaries with both raters [(LRM 1: Gemini 1.5 – Flash- *generated knowledge*: 4; Gemini 1.5 – Pro- *generated knowledge*: 7. GPT – 4o mini- *generated knowledge*: 11; GPT-4-*few-shot*: 6 and Llama 3.1-*zero-shot*: 39); (LRM 2: Gemini 1.5 – Flash- *generated knowledge*: 12; Gemini 1.5 – Pro- *generated knowledge*: 9. GPT – 4o mini- *generated knowledge*: 12; GPT-4-*few-shot*: 11 and Llama 3.1-*zero-shot*: 44)]. Among these disagreements, all were attributed to the human raters marking a summary as “Accurate” while the LRM had marked the same lay summary as “Inaccurate”. There was only one instance, for Llama 3.1, where LRM 1 found a summary to be accurate, while neither of the human raters did. Additionally, for the criteria of conciseness, both LRMs found Gemini 1.5 model-*generated knowledge* lay summaries to be more concise than those from the other models [(LRM 1; Flash: 97% and Pro: 98%) (LRM 2: Flash: 100% and Pro: 100%)].

Finally, for questions comparing the LLM-generated lay summaries with one another, the LRMs found GPT-4 (*few-shot)* lay summaries to contain more important information (LRM 1: 99; LRM 2: 60), and Gemini 1.5 – Flash (*generated knowledge)* lay summaries contained less non-important information (LRM 1: 82; LRM 2: 68) (Fig 4). LRM 1 most often rated GPT-4 (*few-shot)* lay summaries as containing less false information (56), while LRM 2 most frequently selected Gemini 1.5 – Flash (*generated knowledge)* summaries (46).

### Third Mode (Quantitative evaluation of generated lay summaries)

Table 4 contains the readability metrics of the generated lay summaries per model. Gemini 1.5 – Pro with *generated knowledge* produced the most accessible summaries with the highest FRE score of 67.84 and the lowest FKGL of 7.55. In contrast, GPT-4o mini with *generated knowledge* generated lay summaries with the lowest FRE of 53.69 and the highest FKGL of 10.77. The Friedman test found the readability metrics significantly different per model (*P* < 2.36 × 10^-33^). Nemenyi post hoc tests showed that Llama 3.1 *(zero-shot)* performed significantly differently than other models in all readability metrics (*P* < 0.001). The other models demonstrated intermediate readability with grade levels ranging from 8.3 to 9.4. Additionally, based on the average character length, the Gemini 1.5 models with *generated knowledge* prompts generated more concise lay summaries compared to Llama 3.1 and GPT-4. More interestingly, GPT-4 and Llama 3.1 with *generated knowledge* prompts performed worse across 4 out of 5 readability scores,when compared to GPT-4 with *few-shot* and Llama 3.1 with *zero-shot* prompt.

**Table 4:** Summary of readability metrics and the average character length of the generated lay summaries per model.*.

Models (Prompt)	Flesch Reading Ease (↑)	Flesch Kincaid Grade (↓)	SMOG Index (↓)	Automated Readability Index (↓)	Dale- Chall Score (↓)	Average Character Length (↓)
Original Report	30.81 ± 10.62	12.34 ± 1.97	13.98 ± 1.82	12.82 ± 2.38	12.7 ± 0.79	1906.30
Gemini 1.5 – Flash (Generated Knowledge)	57.62 ± 8.85	9.12 ± 1.62	11.80 ± 1.45	10.34 ± 1.92	10.84 ± 0.72	694.33
Gemini 1.5 – Pro (Generated Knowledge)	**67.84 ± 7.78**	**7.55 ± 1.38**	**10.33 ± 1.45**	**8.83 ± 1.62**	10.09 ± 0.80	**568.26**
GPT-4o mini (Generated Knowledge)	53.69 ± 6.92	10.77 ± 1.30	13.35 ± 1.25	12.08 ± 1.62	10.62 ± 0.72	1191.08
GPT-4 (Few-shot)	62.10 ± 7.98	9.41 ± 1.59	12.05 ± 1.36	10.45 ± 1.92	9.94 ± 0.63	1464.89
GPT-4 (Generated Knowledge)	60.46 ± 7.97	9.61 ± 1.50	12.02 ± 1.27	10.84 ± 1.77	10.04 ± 0.69	1414.23
Llama 3.1 (Zero-shot)	65.03 ± 9.97	8.31 ± 1.75	11.28 ± 1.55	9.01 ± 1.92	**9.24 ± 0.98**	1711.89
Llama 3.1 (Generated Knowledge)	57.45 ± 14.00	9.51 ± 2.56	12.35 ± 2.14	9.98 ± 2.99	9.99 ± 1.35	1170.98

* Note – Abbreviations: SMOG = Simple Measure of Gobbledygook. For GPT-4o and Llama 3.1, generated knowledge prompting was added to so that all models can be compared against a single prompting strategy as well, beside their tailored prompting strategies.

### Overall prompt length and lay summary quality

The study found varying relationship between the character length of the “findings” (or prompt length) and the quality of generated lay summaries (S6 Text). Across the LLM-tailored prompts and human raters, longer findings were consistently associated with higher levels of extraneous information (e.g., GPT-4-*few-shot*: ρ ≈ 0.343 - 0.553, *P* < 0.001; GPT-4o mini- *generated knowledge* and Llama 3.1-*zero-shot*: ρ ≈ 0.243–0.343, *P* < 0.01). However, this did not translate into the inclusion of more relevant or actionable information, as demonstrated by significant negative correlations for GPT-4o mini (*generated knowledge)* and GPT-4 (*few-shot)* across both the LRM and human raters (ρ ≈ –0.34 to –0.52, *P* < 0.001). Moreover, accurate lay summaries generated by Llama 3.1 (*zero-shot)* were associated with higher median character counts compared with inaccurate ones (Rater 1: *P* = 0.011; Rater 2: *P* = 0.022), indicating that, for certain models, longer prompts may sometimes enhance accuracy despite the risk of extraneous content. *Generated knowledge* prompts reduced the length of lay summaries from GPT-4 (*few-shot)* and Llama 3.1 *(zero-shot)* compared to their tailored prompt but at the cost of their readability scores.

## Discussion

In this study, we explore five LLMs and tailored prompt combinations, [{Gemini 1.5 – Pro, Gemini 1.5 – Flash and GPT-4o mini (*generated knowledge*)}, GPT-4 (*few-shot*) and Llama 3.1(*zero-shot*)], in the task of generating patient-friendly lay summaries for radiology reports. These tailored prompting techniques (*zero-shot, few-shot* and *generated knowledge*) were used to analyze the impact of prompt design on the LLMs’ performance. We selected radiology (e.g., X-ray, CT) reports to generate lay summaries because these are some of the most common imaging tests and typically, radiology reports have a free-text portion with findings and impressions sections [39]. The study utilizes a comprehensive mixed evaluation framework to evaluate the LLMs. The framework contains human raters’ and two LRMs’ assessment using key subjective criteria (Inaccurate Information, Actionable Information, Beneficial Supplementary Information, Extraneous Information and No Supervision) and quantitative readability metrics (Flesch Reading Ease, Flesch-Kincaid Grade, SMOG Index, Automated Readability Index and Dale-Chall Score) (Fig 1). To the best of our knowledge, this study is the first to tailor prompting techniques uniquely selected for a group of LLMs and to use LRM evaluators to assess lay summaries.

Key findings from this study show that based on percentage agreement ranking of Likert statement responses by human raters and the LRMs (subjective evaluation), Gemini 1.5 – Flash with *generated knowledge* prompt ranked first in producing lay summaries with the least amount of inaccurate information and including the most actionable information. Gemini 1.5 – Pro with *generated knowledge* prompt ranked second but had the most accessible summaries determined through readability metrics (quantitative evaluation), indicating suitability for readers around the 7th to 8th grade level and was the most concise. There was disagreement between the human raters and the LRM raters regarding the LLM that produced the most accurate lay summaries (Human raters: GPT-4-*few-shot*; LRM 1: Gemini 1.5 – Flash-*generated knowledge*; LRM 2: Gemini 1.5 – Pro- *generated knowledge*).

Additionally, in contrast to the demonstrated superiority of GPT-4, in previous literature, for lay summary generation tasks from radiology reports, our study highlights the Gemini 1.5 family models. We found that Gemini 1.5 – Flash and Gemini 1.5 – Pro with the *generated knowledg*e prompt outperformed other models including GPT-4 with a *few-shot* and *generated knowledge* prompts in generating lay summaries, according to both subjective and quantitative evaluations.

### Simplification and improved readability across all LLMs

There was an overall improvement in readability and simplification of the original report by all LLMs. The original reports reflected a Grade 12 and above reading level required for comprehension. In comparison, all LLM-generated lay summaries were substantially more readable as demonstrated by the higher FRE and lower grade-level metrics across all LLMs. It is possible that the conciseness of lay summaries, in comparison to that of the original reports, improved the readability scores for the LLMs-generated summaries.

### Gemini 1.5 model performance and prompting technique

Gemini 1.5 – Flash and Gemini 1.5 – Pro with *generated knowledge* prompts consistently outperformed other model-prompt combinations in all Likert criteria, across both human and LRM evaluations. The *generated knowledge* prompt might have benefited the performance for these specific models (“Consider what a good lay summary should have. Apply these considerations into generating a lay summary.”) as the LLMs were asked to use their foundational knowledge of lay summaries instead of relying on detailed examples. The LLMs likely retrieved latent knowledge about lay communication and internalized the principles of lay summaries before generating the outputs [8]. This highlights the combined superiority of the Gemini 1.5 models with *generated knowledge* prompts in generating lay summaries.

### GPT-4 performance and prompting technique

Although GPT-4 had previously demonstrated top performance in the literature using *zero-shot* for the lay summarization task, it showed comparatively lower performance under *few-shot* and *generated knowledge* prompting against the Gemini models with *generated knowledge* prompts in our study [9–13]. It ranked third or fourth in the percentage agreement ranking of human and LRM evaluations and had the highest average character length (1,464.89). Despite this, the GPT-4 (*few-shot)* summaries were rated highly for accuracy (98% by human raters; 94% by LRM 1; 88% by LRM 2). One explanation for the results could be the use of *few-shot* prompting with examples provided within the prompt to guide the LLM. It is possible that the use of examples constrained the model and the outputs “overfit” to the examples. And yet we found from the pilot study (Table A in S2 Text), based on three iterations of various prompt styles, that GPT-4 had a significant suitability to the *few-shot* prompt over any other prompt styles including *generated knowledge* and GPT-4 with *generated knowledge* did not perform as well quantitatively, as the model under *few-shot* prompt (Table 4).

### No Supervision criteria

For the “ No Supervision” criteria – where the raters judged whether the lay summaries could be shared directly with patients without any supervision - Gemini 1.5 Pro (*generated knowledge)* received the highest agreement scores (Human Raters: 71%, Human Raters and LRM 61%), followed by Gemini 1.5 – Flash with *generated knowledge* prompt (Human Raters: 70%, Human Raters and LRM 60%), compared to other model-prompt configurations. While this is a significant feat as it indicates that Gemini 1.5 family model summaries with *generated knowledge* prompts were more likely to be perceived as trustworthy, accurate and self-sufficient, the remaining ~30% of summaries raises concern and suggests a need for stronger safety checks on LLM-generated summaries. The use of this evaluation criterion and subsequent results can have potential implications in real-life clinical workflow research.

### Gemini 2.5 – Pro vs GPT-oss-120b (LRMs) performance as an evaluator

The LRMs showed strong alignment with the human raters for percentage agreement ranking although GPT-oss-120b had a higher percentage difference from human raters (<30%) in Likert scores across all models and criteria compared to Gemini 2.5 – Pro (<11%). Additionally, Gemini 2.5 – Pro had a smaller complete disagreement (0.96% vs 3.4%) and partial disagreement (25% vs 36%) with the human raters on Likert criteria than GPT-oss-120b. The LRMs were slightly more conservative than the human raters when labelling summaries as accurate and were more likely to label summaries as “Inaccurate” even when the human raters labelled them as “Accurate”. Overall, the LRM raters had 72% agreement (1818/2500 responses). Both the LRMs also independently ranked Gemini 1.5 family models-*generated knowledge* prompts as first and second, and found them to be more accurate. Despite being an OpenAI model, GPT-oss-120b did not preferentially rate its own family models’ (GPT-4-*few-shot* or GPT-4o mini- *generated* *knowledge*) summaries higher than those produced by other LLMs. This cross-family agreement suggests that the Gemini 2.5 – Pro’s high ranking of Gemini family models is unlikely to be driven by model self-preference [35]. These findings highlight promising yet preliminary potential of LRMs as scalable, second-pass reviewers in the clinical context, that maybe capable of validating the quality and accuracy of lay summaries for patients, however further external validation is needed on larger cohorts and to explore any potential shared model biases.

### Interpretation of findings in the current AI landscape

Newer versions of the evaluated models (e.g., GPT-5 and Llama 4) have been released since the completion of this study. While these newer versions may potentially affect overall performance in lay summary generation, the objective of the study was to explore these methodologies and evaluation frameworks, and to identify the limitations and strengths of the LLM-prompt combinations, rather than strictly perform LLM benchmarking. The methodological insights from this study such as the prompt templates, comprehensive LRM evaluation framework and/or the pilot study designs, are expected to remain applicable to newer models and relevant to researchers and clinicians. Additionally, the results from the LLMs used in this study should be interpreted as a snapshot-in-time evaluation of older versions and can be used as a reference to show model performance change of newer versions.

### Low to fair inter-rater human agreement

There was an overall low to fair inter-rater human agreement, but this did not largely impact the results as there was only 8.5% of polarizing opinions (Agree vs. Disagree) between the two human raters. The inter-rater agreement was the result of conservative calculations by measuring agreement across all 5-point Likert scores (Strongly Agree, Agree, Neutral, Disagree and Strongly Disagree) rather than aggregating the ratings into 3 scores (Agree, Neutral and Disagree). Since one rater was more likely to use the Neutral option, this impacted the inter-rater agreement. Therefore, to interpret the results, the median ratings for Likert statements and the average ratings from both human raters when comparing against the LRMs were used.

### Future directions and recommendations

The study results add to the growing body of literature on the summary generation capabilities of LLMs and highlight the potential of using LRMs as evaluators. The study also found that the prompt technique impacts the output and directly compared all prompt styles with the LLMs using quantitative readability metrics. However, direct comparability using expert and LRM evaluation, between the LLMs and prompt style was out of scope for this study and therefore, an in-depth exploration of prompting techniques per model is required for performance evaluation. Additionally, given the “No Supervision” criteria results, the LLMs and LRM could be implemented in the radiology workflow with some degree of automation but with strong caution and incorporation of strict radiologist oversight workflow [40]. Further research is needed with (1) increased expert participation and (2) to explore the “patient-friendliness” (patient comprehension) and readability of LLM-generated lay summaries, from the perspective of the patient.

### Limitations

This study has some limitations. Firstly, there was a low-to-fair inter-rater agreement among the experts (S4 Text). Second, the study had a small sample size of 100 summaries with two human raters and two LRMs which could limit generalizability. While the 100 radiology reports were sampled from varying character counts, the small sample size may not have captured very long reports with rare pathologies that could potentially lead to model hallucinations. Third, a pilot study was used to select the prompts based on the highest median cosine similarity (objective metric) between human generated lay summaries and abstracts from the Cochrane Database of Systematic Reviews (S2 Text). It is possible that the use of only one objective metric and non-radiology related dataset with a ground truth of subjective nature, may have introduced a potential bias by highlighting a prompt that does not fully capture the summarization goals of a radiology report. Therefore to mitigate this, all prompts were designed with the same goal (“The goal is to simplify the radiology report for non-experts and make the report easily accessible and understandable for the broader public, patient and patient’s family”). Additionally in the evaluation metric, while we had a criterion for Beneficial Supplementary Information, it did not rule out the presence of harmful information. The raters did not evaluate directly for inclusion of harmful information but there were safety checks in place (such as Inaccurate Information). It is unclear if the “inaccurate” information flagged by raters was potentially dangerous or benign as the scope of the project did not allow for follow-up questions to the raters. Another limitation is that the readability metrics included in this study only assessed syntactic complexity (sentence length/syllables) rather than semantic difficulty which may not fully capture layperson understanding and true patient comprehension. Also, our study is limited to the reports contained in our sample dataset. A more nuanced analysis based on disease type and imaging modality could not be performed, as those are not provided in the dataset. However, we have used length of reports as a surrogate of complexity and found that LLMs vary in accuracy and in producing extraneous content with longer findings. The LRMs chosen are from Gemini and GPT family and could be affected by self-preference bias, but in our experiments, GPT-oss-120B did not demonstrate such a preference as it indicated a Gemini model to be better than models from the GPT family for most Likert criteria and accuracy. Finally, this study is a result of single-run iteration where multiple runs could have potentially improved the results. Although fixed prompts and hyperparameters were used to improve consistency, some variation in phrasing, readability, and content emphasis would be expected across repeated generations, which may affect reproducibility of the reported results.

## Conclusion

In conclusion, this study presents a comprehensive evaluation of LLMs with selected prompts in generating lay summaries from radiology reports, using a mixed-evaluation (subjective and objective) framework. Gemini 1.5 – Flash and Gemini 1.5 – Pro using *generated knowledge* prompt outperformed other model-prompt combinations in generating lay summaries across readability metrics, Likert ratings, and the “No Supervision” criterion. The LRM raters showed strong alignment with each other and human raters, however Gemini 2.5 – Pro (LRM 1) had higher agreement and lower polarizing disagreements with human raters across most evaluation criteria, compared to GPT-oss-120b (LRM 2) highlighting its performance as an evaluator. Additionally, GPT-4 under the *few-shot* prompting style produced the most accurate lay summaries according to the human raters. In view of the fast-evolving nature of AI models, findings from this study builds methodological insights, shows potential in pairing appropriate prompting styles with any LLM and application of mixed evaluation frameworks, when using LLMs for clinical communication, rather than serving as a performance benchmark.

## Supporting information

S1 Text Pilot Study 1. (DOCX)

S2 Text Pilot Study 2. (DOCX)

S3 Text Expert and LRM evaluation questionnaire. (DOCX)

S4 Text Inter-rater reliability metric. (DOCX)

S5 Text Percentage Agreement Rankings. (DOCX)

S6 Text Statistical significance between length of findings section and lay summary quality per rater. (DOCX)

S1 Fig Agreement matrices showing count comparisons between Human Raters 1 and 2 for each Large Language Model and Likert criteria. (TIF)

S1 Data Evaluation responses of all raters (Gemini 2.5 - Pro, GPT-oss-120b, Human Rater 1 and 2) of the lay summaries per LLM. (XLSX)
