# Supplementary material for: Evaluating large language models for lay summaries of radiology reports using tailored prompting strategies and mixed-method assessment
Source: PLOS Digit Health. 2026 Sep 17;5(9):e0001672. doi: 10.1371/journal.pdig.0001672 (PMC13585270; doi:10.1371/journal.pdig.0001672)
Supplement: Supplementary file 1 [file pdig.0001672.s001.docx]

## **S1 Text. Pilot Study 1**

**Objectives**

To select LLMs for the main experiment of lay summary generation.

**Methods**

Multiple LLMs were prompted with the following prompt:

“Please produce a lay summary of this radiology report: <SAMPLE RADIOLOGY REPORT>”

Various reading metrics of the output including Flesch-Kincaid Grade Level (FKGL), Gunning Fog Index, Coleman-Liau Index and Automated Readability Index, were calculated.

**Results**

Table A contains the results of the LLMs and reading metrics. The rationale for the selected LLMs (Gemini 1.5 – Pro, Gemini 1.5 – Flash, GPT-4o mini and Llama 3.1) include FKGL value of 8-10^th^ grade level, summary quality, LLMs’ recent release (2024), identified literature gap and availability of Application Programming Interface (API). GPT-4 was selected based on its reported high performance in multiple studies in text generation tasks and availability of API.

**Table A: Pilot study 1 readability metric results of LLMs.**

| Model | Flesch -Kincaid Grade Level | Gunning Fog Index | Coleman-Liau Index | Automated Readability Index |
| --- | --- | --- | --- | --- |
| GPT-4 | 10.7 | 10.67 | 10.37 | 13.7 |
| GPT-3.5 | 6.8 | 8.851 | 8.98 | 5.31 |
| Gemini 1.5 – Flash | 9.78 | 12.00 | 9.51 | 8.80 |
| Gemini 1.5 – Pro | 8.47 | 8.86 | 9.83 | 7.59 |
| Microsoft Copilot | 9.1 | 10.43 | 12.0 | 11.2 |
| Facebook BART | 5.7 | 8.01 | 12.21 | 9.3 |
| Meta Llama 3.1 | 8.86 | 11.10 | 10.56 | 7.53 |
| Med Alpaca | 8.2 | 9.29 | 9.61 | 8.1 |
| Google Pegasus | 8.2 | 10.12 | 12.03 | 9.9 |
| Meditron | 5.5 | 8.08 | 2.95 | 0.66 |

**Conclusion**

The LLMs selected for the main experiment include Gemini 1.5 – Pro, Gemini 1.5 – Flash, GPT-4o mini, GPT-4 and Llama 3.1.
