# Supplementary material for: Evaluating large language models for lay summaries of radiology reports using tailored prompting strategies and mixed-method assessment
Source: PLOS Digit Health. 2026 Sep 17;5(9):e0001672. doi: 10.1371/journal.pdig.0001672 (PMC13585270; doi:10.1371/journal.pdig.0001672)
Supplement: Supplementary file 2 [file pdig.0001672.s002.docx]

## **S2 Text. Pilot Study 2**

**Objective**

To determine the best performing prompt and combination of hyperparameters (Top_p and temperature) with the highest median cosine similarity score for text summarization task by 5 Large Language Models (LLMs) (Gemini 1.5 – Pro, Gemini 1.5 – Flash, GPT-4o mini, GPT-4 and Llama 3.1).

**Methods**

This study was conducted on a subset of the Cochrane Database of Systematic Reviews dataset^1^. This dataset was used because it contained high-quality abstracts of systematic reviews from the health care domain with medical jargon, had the same summarization demands as radiology report patient summaries and contained human expert generated lay summaries (ground truth). There are currently no publicly available radiology report datasets that also contain ground truth lay summaries generated by experts.

On a total of 10 studies, seven types of prompt techniques were used and they have been provided below:

Zero-shot role-based: “Act as an educator and write a lay summary using the study abstract. The goal is to simplify the abstract for non-experts and make the study easily accessible and understandable for the broader public. The summary should be written in plain English so that it understandable at different reading levels. Generate a title for the summary. Avoid jargon and acronyms. Explain any complex terminology if important to the lay summary. Create simple sentences without many clauses. Use an active voice. Use clear and concise language. Avoid ambiguity. Write the lay summary in a logical manner. Organize lay summary in sections where appropriate. Comment on the quality of evidence."

Zero-shot with no role: “Write a lay summary using the study abstract. The goal is to simplify the abstract for non-experts and make the study easily accessible and understandable for the broader public. The summary should be written in plain English so that it understandable at different reading levels. Generate a title for the summary. Avoid jargon and acronyms. Explain any complex terminology if important to the lay summary. Create simple sentences without many clauses. Use an active voice. Use clear and concise language. Avoid ambiguity. Write the lay summary in a logical manner. Organize lay summary in sections where appropriate. Comment on the quality of evidence."

Few-shot role-based: “Act as an educator and write a lay summary using the study abstract. The goal is to simplify the abstract for non-experts and make the study easily accessible and understandable for the broader public. The summary should be written in plain English so that it understandable at different reading levels. Generate a title for the summary. Avoid jargon and acronyms. Explain any complex terminology if important to the lay summary. Create simple sentences without many clauses. Use an active voice. Use clear and concise language. Avoid ambiguity. Write the lay summary in a logical manner. Organize lay summary in sections where appropriate. Comment on the quality of evidence. Use the example as a guide: {Abstract: “Background About 10% of reproductive‐aged women suffer from endometriosis, which is a costly, chronic disease that causes pelvic pain and subfertility. Laparoscopy is the gold standard diagnostic test for endometriosis, but it is expensive and carries surgical risks. Currently, there are no non‐invasive tests available in clinical practice that accurately diagnose endometriosis. This is the first diagnostic test accuracy review of endometrial biomarkers for endometriosis that utilises Cochrane methodologies, providing an update on the rapidly expanding literature in this field. Objectives To determine the diagnostic accuracy of the endometrial biomarkers for pelvic endometriosis, using a surgical diagnosis as the reference standard. We evaluated the tests as replacement tests for diagnostic surgery and as triage tests to inform decisions to undertake surgery for endometriosis. Search methods We did not restrict the searches to particular study designs, language or publication dates. To identify trials, we searched the following databases: CENTRAL (2015, July), MEDLINE (inception to May 2015), EMBASE (inception to May 2015), CINAHL (inception to April 2015), PsycINFO (inception to April 2015), Web of Science (inception to April 2015), LILACS (inception to April 2015), OAIster (inception to April 2015), TRIP (inception to April 2015) and ClinicalTrials.gov (inception to April 2015). We searched DARE and PubMed databases up to April 2015 to identify reviews and guidelines as sources of references to potentially relevant studies. We also performed searches for papers recently published and not yet indexed in the major databases. The search strategies incorporated words in the title, abstract, text words across the record and the medical subject headings (MeSH). Selection criteria We considered published peer‐reviewed, randomised controlled or cross‐sectional studies of any size that included prospectively collected samples from any population of reproductive‐aged women suspected of having one or more of the following target conditions: ovarian, peritoneal or deep infiltrating endometriosis (DIE). Data collection and analysis Two authors independently extracted data from each study and performed a quality assessment. For each endometrial diagnostic test, we classified the data as positive or negative for the surgical detection of endometriosis and calculated the estimates of sensitivity and specificity. We considered two or more tests evaluated in the same cohort as separate data sets. We used the bivariate model to obtain pooled estimates of sensitivity and specificity whenever sufficient data were available. The predetermined criteria for a clinically useful test to replace diagnostic surgery was one with a sensitivity of 94% and a specificity of 79%. The criteria for triage tests were set at sensitivity at or above 95% and specificity at or above 50%, which in case of negative results rules out the diagnosis (SnOUT test) or sensitivity at or above 50% with specificity at or above 95%, which in case of positive result rules in the diagnosis (SpIN test). Main results We included 54 studies involving 2729 participants, most of which were of poor methodological quality. The studies evaluated endometrial biomarkers either in specific phases of the menstrual cycle or outside of it, and the studies tested the biomarkers either in menstrual fluid, in whole endometrial tissue or in separate endometrial components. Twenty‐seven studies evaluated the diagnostic performance of 22 endometrial biomarkers for endometriosis. These were angiogenesis and growth factors (PROK‐1), cell‐adhesion molecules (integrins α3β1, α4β1, β1 and α6), DNA‐repair molecules (hTERT), endometrial and mitochondrial proteome, hormonal markers (CYP19, 17βHSD2, ER‐α, ER‐β), inflammatory markers (IL‐1R2), myogenic markers (caldesmon, CALD‐1), neural markers (PGP 9.5, VIP, CGRP, SP, NPY, NF) and tumour markers (CA‐125). Most of these biomarkers were assessed in single studies, whilst only data for PGP 9.5 and CYP19 were available for meta‐analysis. These two biomarkers demonstrated significant diversity for the diagnostic estimates between the studies; however, the data were too limited to reliably determine the sources of heterogeneity. The mean sensitivities and specificities of PGP 9.5 (7 studies, 361 women) were 0.96 (95% confidence interval (CI) 0.91 to 1.00) and 0.86 (95% CI 0.70 to 1.00), after excluding one outlier study, and for CYP19 (8 studies, 444 women), they were were 0.77 (95% CI 0.70 to 0.85) and 0.74 (95% CI 0.65 to 84), respectively. We could not statistically evaluate other biomarkers in a meaningful way. An additional 31 studies evaluated 77 biomarkers that showed no evidence of differences in expression levels between the groups of women with and without endometriosis. Authors' conclusions We could not statistically evaluate most of the biomarkers assessed in this review in a meaningful way. In view of the low quality of most of the included studies, the findings of this review should be interpreted with caution. Although PGP 9.5 met the criteria for a replacement test, it demonstrated considerable inter study heterogeneity in diagnostic estimates, the source of which could not be determined. Several endometrial biomarkers, such as endometrial proteome, 17βHSD2, IL‐1R2, caldesmon and other neural markers (VIP, CGRP, SP, NPY and combination of VIP, PGP 9.5 and SP) showed promising evidence of diagnostic accuracy, but there was insufficient or poor quality evidence for any clinical recommendations. Laparoscopy remains the gold standard for the diagnosis of endometriosis, and using any non‐invasive tests should only be undertaken in a research setting. We have also identified a number of biomarkers that demonstrated no diagnostic value for endometriosis. We recommend that researchers direct future studies towards biomarkers with high diagnostic potential in good quality diagnostic studies. Lay Summary: Endometrial biomarkers for the non‐invasive diagnosis of endometriosis Review question Can physicians use biomarkers (distinctive molecules, genes or other characteristics that appear in certain conditions) to reduce the need to surgically diagnose endometriosis? Background The endometrium refers to the tissue that lines the womb and is shed during menstruation. Women with endometriosis have endometrial tissue growing outside the womb, within the pelvic cavity. This tissue responds to reproductive hormones causing painful periods, chronic lower abdominal pain and difficulty conceiving. Currently the only reliable way of diagnosing endometriosis is to perform keyhole surgery and visualise the endometriotic deposits inside the abdomen. Because surgery is risky and expensive, various tests within the endometrium that can be obtained during an in‐office womb sampling procedure have been assessed for their ability to detect endometriosis non‐invasively or with minimal invasion. An accurate test could lead to the diagnosis of endometriosis without the need for surgery, or it could reduce the need for diagnostic surgery so only women who were most likely to have endometriosis would require it. Review teams have also evaluated other non‐invasive ways of diagnosing endometriosis using blood, urine and imaging tests as well as a combination of several testing methods in separate Cochrane reviews within this series. Study characteristics The evidence in this review is current to April 2015. We included 54 studies involving 2729 participants. All studies evaluated reproductive‐aged women who were undertaking diagnostic surgery to investigate symptoms of endometriosis or for other indications. Twenty‐six studies evaluated the role of 22 different biomarkers in diagnosing endometriosis, and 31 studies identified 77 additional biomarkers that had no value in differentiating between women with and without the disease. Key results and quality of evidence Only two of the assessed biomarkers, a neural fibre marker PGP 9.5 and hormonal marker CYP19, were assessed in sufficient number of studies to obtain meaningful results. PGP 9.5 identified endometriosis with enough accuracy to replace surgical diagnosis. Several additional biomarkers (endometrial proteome, 17βHSD2, IL‐1R2, caldesmon and other neural markers) show promise in detecting endometriosis, but there are too few studies to be sure of their diagnostic value. The studies differed in how they were conducted, which groups of women were studied and how the surgery was undertaken. The reports were of low methodological quality, which is why readers cannot consider these results to be reliable unless confirmed in large, high quality studies. Overall, there is not enough evidence to recommend any endometrial test for use in clinical practice for the diagnosis of endometriosis. Future research Further high quality research is necessary to accurately evaluate the diagnostic potential of the endometrial biomarkers for the diagnosis of endometriosis.”}{“Abstract: Background In order to prevent overweight and obesity in the general population we need to understand the relationship between the proportion of energy from fat and resulting weight and body fatness in the general population. Objectives To assess the effects of proportion of energy intake from fat on measures of weight and body fatness (including obesity, waist circumference and body mass index) in people not aiming to lose weight, using all appropriate randomised controlled trials (RCTs) and cohort studies in adults, children and young people Search methods We searched CENTRAL to March 2014 and MEDLINE, EMBASE and CINAHL to November 2014. We did not limit the search by language. We also checked the references of relevant reviews. Selection criteria Trials fulfilled the following criteria: 1) randomised intervention trial, 2) included children (aged ≥ 24 months), young people or adults, 3) randomised to a lower fat versus usual or moderate fat diet, without the intention to reduce weight in any participants, 4) not multifactorial and 5) assessed a measure of weight or body fatness after at least six months. We also included cohort studies in children, young people and adults that assessed the proportion of energy from fat at baseline and assessed the relationship with body weight or fatness after at least one year. We duplicated inclusion decisions and resolved disagreement by discussion or referral to a third party. Data collection and analysis We extracted data on the population, intervention, control and outcome measures in duplicate. We extracted measures of weight and body fatness independently in duplicate at all available time points. We performed random‐effects meta‐analyses, meta‐regression, subgrouping, sensitivity and funnel plot analyses. Main results We included 32 RCTs (approximately 54,000 participants) and data from 25 cohorts. There is consistent evidence from RCTs in adults of a small weight‐reducing effect of eating a smaller proportion of energy from fat; this was seen in almost all included studies and was highly resistant to sensitivity analyses. The effect of eating less fat (compared with usual diet) is a mean weight reduction of 1.5 kg (95% confidence interval (CI) ‐2.0 to ‐1.1 kg), but greater weight loss results from greater fat reductions. The size of the effect on weight does not alter over time and is mirrored by reductions in body mass index (BMI) (‐0.5 kg/m 2 , 95% CI ‐0.7 to ‐0.3) and waist circumference (‐0.3 cm, 95% CI ‐0.6 to ‐0.02). Included cohort studies in children and adults most often do not suggest any relationship between total fat intake and later measures of weight, body fatness or change in body fatness. However, there was a suggestion that lower fat intake was associated with smaller increases in weight in middle‐aged but not elderly adults, and in change in BMI in the highest validity child cohort. Authors' conclusions Trials where participants were randomised to a lower fat intake versus usual or moderate fat intake, but with no intention to reduce weight, showed a consistent, stable but small effect of low fat intake on body fatness: slightly lower weight, BMI and waist circumference compared with controls. Greater fat reduction and lower baseline fat intake were both associated with greater reductions in weight. This effect of reducing total fat was not consistently reflected in cohort studies assessing the relationship between total fat intake and later measures of body fatness or change in body fatness in studies of children, young people or adults. Lay Summary: Effect of cutting down the fat we eat on body weight The ideal proportion of energy from fat in our food and its relation to body weight is not clear. This review looked at the effect of cutting down the proportion of energy from fat in our food on body weight and fatness in both adults and children who are not aiming to lose weight. The review found that cutting down on the proportion of fat in our food leads to a small but noticeable decrease in body weight, body mass index and waist circumference. This effect was found both in adults and children. The effect did not change over time.”}" [ Examples added from the Cochrane Database of Systematic Reviews^1^]

Chain of Thought with zero-shot: “Act as an educator and write a lay summary using the study abstract. The goal is to simplify the abstract for non-experts and make the study easily accessible and understandable for the broader public. The summary should be written in plain English so that it understandable at different reading levels. Generate a title for the summary. Avoid jargon and acronyms. Explain any complex terminology if important to the lay summary. Create simple sentences without many clauses. Use an active voice. Use clear and concise language. Avoid ambiguity. Write the lay summary in a logical manner. Organize lay summary in sections where appropriate. Comment on the quality of evidence. Lets solve this step by step: What steps are you taking to generate a lay summary from this abstract? Once you have considered the question apply these steps into generating the lay summary.”

Chain of Thought with few-shot: “Act as an educator and write a lay summary using the study abstract. The goal is to simplify the abstract for non-experts and make the study easily accessible and understandable for the broader public. The summary should be written in plain English so that it understandable at different reading levels. Generate a title for the summary. Avoid jargon and acronyms. Explain any complex terminology if important to the lay summary. Create simple sentences without many clauses. Use an active voice. Use clear and concise language. Avoid ambiguity. Write the lay summary in a logical manner. Organize lay summary in sections where appropriate. Comment on the quality of evidence. Lets solve this step by step: What steps are you taking to generate a lay summary from this abstract? Once you have considered the question apply these steps into generating the lay summary. Use the following example as a guide: {“ARTICLE In subfertile couples, couples who have tried to conceive for at least one year, intrauterine insemination (IUI) with ovarian hyperstimulation (OH) is one of the treatment modalities that can be offered. When IUI is performed a second IUI in the same cycle might add to the chances of conceiving. In a previous update of this review in 2010 it was shown that double IUI increases pregnancy rates when compared to single IUI. Since 2010, different clinical trials have been published with differing conclusions about whether double IUI increases pregnancy rates compared to single IUI. Objectives To determine the effectiveness and safety of double intrauterine insemination (IUI) compared to single IUI in stimulated cycles for subfertile couples. Search methods We searched the Cochrane Gynaecology and Fertility (CGF) Group trials register, CENTRAL, MEDLINE, Embase and CINAHL in July 2020 and LILACS, Google scholar and Epistemonikos in February 2021, together with reference checking and contact with study authors and experts in the field to identify additional studies. Selection criteria We included randomised controlled, parallel trials of double versus single IUIs in stimulated cycles in subfertile couples. Data collection and analysis Two authors independently assessed trial quality and extracted data. We contacted study authors for additional information. Main results We identified in nine studies involving subfertile women. The evidence was of low quality; the main limitations were unclear risk of bias, inconsistent results for some outcomes and imprecision, due to small trials with imprecise results. We are uncertain whether double IUI improves live birth rate compared to single IUI (odds ratio (OR) 1.15, 95% confidence interval (CI) 0.71 to 1.88; I 2 = 29%; studies = 3, participants = 468; low quality evidence). The evidence suggests that if the chance of live birth following single IUI is 16%, the chance of live birth following double IUI would be between 12% and 27%. Performing a sensitivity analysis restricted to only randomised controlled trials (RCTs) with low risk of selection bias showed similar results. We are uncertain whether double IUI reduces miscarriage rate compared to single IUI (OR 1.78, 95% CI 0.98 to 3.24; I 2 = 0%; studies = 6, participants = 2363; low quality evidence). The evidence suggests that chance of miscarriage following single IUI is 1.5% and the chance following double IUI would be between 1.5% and 5%. The reported clinical pregnancy rate per woman randomised may increase with double IUI group (OR 1.51, 95% CI 1.23 to 1.86; I 2 = 34%; studies = 9, participants = 2716; low quality evidence). This result should be interpreted with caution due to the low quality of the evidence and the moderate inconsistency. The evidence suggests that the chance of a pregnancy following single IUI is 14% and the chance following double IUI would be between 16% and 23%. We are uncertain whether double IUI affects multiple pregnancy rate compared to single IUI (OR 2.04, 95% CI 0.91 to 4.56; I 2 = 8%; studies = 5; participants = 2203; low quality evidence). The evidence suggests that chance of multiple pregnancy following single IUI is 0.7% and the chance following double IUI would be between 0.85% and 3.7%. We are uncertain whether double IUI has an effect on ectopic pregnancy rate compared to single IUI (OR 1.22, 95% CI 0.35 to 4.28; I 2 = 0%; studies = 4, participants = 1048; low quality evidence). The evidence suggests that the chance of an ectopic pregnancy following single IUI is 0.8% and the chance following double IUI would be between 0.3% and 3.2%. Authors' conclusions Our main analysis, of which the evidence is low quality, shows that we are uncertain if double IUI improves live birth and reduces miscarriage compared to single IUI. Our sensitivity analysis restricted to studies of low risk of selection bias for both outcomes is consistent with the main analysis. Clinical pregnancy rate may increase in the double IUI group, but this should be interpreted with caution due to the low quality evidence. We are uncertain whether double IUI has an effect on multiple pregnancy rate and ectopic pregnancy rate compared to single IUI. ELEMENT EXTRACTION: The important entities in this study are single intrauterine insemination, double intrauterine insemination and subfertile couples. The central purpose of the document is to determine the effectiveness and safety of double intrauterine insemination (IUI) compared to single IUI in stimulated cycles for subfertile couples The important date in this study in this study is the year 2010 The events happening in this document are that researchers reviewed 9 studies to determine the effectiveness of double intrauterine insemination (IUI) compared to single IUI on pregnancy outcomes such as chance of single and multiple pregnancy, chance of miscarriage, live birth and chance of Ectopic pregnancy. The result of these events are that the current evidence is of low quality because of potential risk of bias, inconsistent results, small trial size with imprecise results. It is also unclear whether double IUI has an effect on multiple pregnancy rate, ectopic pregnancy rate, miscarriage and live birth rate compared to single IUI. The take-home message based on the results is that there is lack of evidence to support that double intrauterine insemination improved pregnancy outcomes compared to single intrauterine insemination.” “Lay Summary: Double versus single intrauterine insemination for subfertile couples Review question : Cochrane authors reviewed the evidence about the effect of double intrauterine insemination (IUI) versus single IUI in subfertile couples (couples who have tried to conceive for at least one year). Background : for couples who have tried to conceive for at least one year a common way to induce pregnancy is placement of the sperm directly into the uterus and therefore close to any eggs. This is combined with fertility medicines to stimulate the release of eggs (IUI with ovarian stimulation). The insemination is less stressful, invasive and expensive compared to in vitro fertilisation (where an egg is combined with sperm outside the body) and similar procedures. It is often used when a male partner is subfertile, or when the reason for not becoming pregnant is unknown. Generally, IUI is carried out once in a menstrual cycle, but it is sometimes attempted twice (double IUI). Different clinical trials reached differing conclusions whether double IUI resulted in more pregnancies than single IUI. Study characteristics : we found nine randomised controlled trials (clinical studies where people are randomly put into one of two or more treatment groups) comparing double IUI with single IUI with 2751 woman. The evidence is current to July 2020. Key results : our main analysis, of which the evidence is rated as low quality, shows that we are uncertain if double IUI improves live birth and reduces miscarriage compared to single IUI. The evidence suggests that if the chance of live birth following single IUI is 16%, then the chance following double IUI would be between 12% and 27%. The evidence suggests that if chance of miscarriage following single IUI is 1.5%, the chance following double IUI would be between 1.5% and 5%. Performing analysis with the highest quality trials showed similar results for both outcomes. Pregnancy rate may increase with double IUI. This result should be interpreted with caution due to low quality of the evidence. The evidence suggests that chance of pregnancy following single IUI is 14% and the chance following double IUI would be between 16% and 23%. However, when we analysed only with the high-quality studies, the positive effect of double IUI was lost and we no longer saw the improvement anymore. We are uncertain whether double IUI reduces multiple‐ (two or more fetuses) and ectopic pregnancy rate (where a fertilised egg implants itself outside of the womb, usually in one of the tubes connecting the ovary and womb) compared to single IUI. The evidence suggests that if the chance of multiple pregnancy following single IUI is 0.7%, then the chance following double IUI would be between 0.7% and 3.2%. The evidence suggests that if the chance of ectopic pregnancy following single IUI is 0.8% and the chance following double IUI would be between 0.3% and 3.2%. Quality of the evidence : the evidence was of low quality. The main limitations in the evidence were unclear risk of bias and small trials with imprecise results. }{“ARTICLE Background The application of continuous positive airway pressure (CPAP) has been shown to have some benefits in the treatment of preterm infants with respiratory distress. CPAP has the potential to reduce lung damage, particularly if applied early before atelectasis has occurred. Early application may better conserve an infant's own surfactant stores and consequently may be more effective than later application. Objectives • To determine if early compared with delayed initiation of CPAP results in lower mortality and reduced need for intermittent positive‐pressure ventilation in preterm infants in respiratory distress ○ Subgroup analyses were planned a priori on the basis of weight (with subdivisions at 1000 grams and 1500 grams), gestation (with subdivisions at 28 and 32 weeks), and according to whether surfactant was used ▫ Sensitivity analyses based on trial quality were also planned ○ For this update, we have excluded trials using continuous negative pressure Search methods We used the standard search strategy of Cochrane Neonatal to search the Cochrane Central Register of Controlled Trials (CENTRAL; 2020, Issue 6), in the Cochrane Library; Ovid MEDLINE(R) and Epub Ahead of Print, In‐Process & Other Non‐Indexed Citations Daily and Versions(R); and the Cumulative Index to Nursing and Allied Health Literatue (CINAHL), on 30 June 2020. We also searched clinical trials databases and the reference lists of retrieved articles for randomised controlled trials (RCTs) and quasi‐RCTs. Selection criteria We included trials that used random or quasi‐random allocation to either early or delayed CPAP for spontaneously breathing preterm infants in respiratory distress. Data collection and analysis We used the standard methods of Cochrane and Cochrane Neonatal, including independent assessment of trial quality and extraction of data by two review authors. We used the GRADE approach to assess the certainty of evidence. Main results We found four studies that recruited a total of 119 infants. Two were quasi‐randomised, and the other two did not provide details on the method of randomisation or allocation used. None of these studies used blinding of the intervention or the outcome assessor. Evidence showed uncertainty about whether early CPAP has an effect on subsequent use of intermittent positive‐pressure ventilation (IPPV) (typical risk ratio (RR) 0.77, 95% confidence interval (CI) 0.43 to 1.38; typical risk difference (RD) ‐0.08, 95% CI ‐0.23 to 0.08; I² = 0%, 4 studies, 119 infants; very low‐certainty evidence) or mortality (typical RR 0.93, 95% CI 0.43 to 2.03; typical RD ‐0.02, 95% CI ‐0.15 to 0.12; I² = 33%, 4 studies, 119 infants; very low‐certainty evidence). The outcome 'failed treatment' was not reported in any of these studies. There was an uncertain effect on air leak (pneumothorax) (typical RR 1.09, 95% CI 0.39 to 3.04, I² = 0%, 3 studies, 98 infants; very low‐certainty evidence). No trials reported intraventricular haemorrhage or necrotising enterocolitis. No cases of retinopathy of prematurity were reported in one study (21 infants). One case of bronchopulmonary dysplasia was reported in each group in one study involving 29 infants. Long‐term outcomes were not reported. Authors' conclusions All four small trials included in this review were performed in the 1970s or the early 1980s, and we are very uncertain whether early application of CPAP confers clinical benefit in the treatment of respiratory distress, or whether it is associated with any adverse effects. Further trials should be directed towards establishing the appropriate level of CPAP and the timing and method of administration of surfactant when used along with CPAP.” “ELEMENT EXTRACTION The important entities in this study are continuous positive airway pressure (CPAP), preterm infants and respiratory distress. The central purpose of the document is to determine if early compared with delayed initiation of CPAP results in lower mortality and reduced need for intermittent positive‐pressure ventilation in preterm infants in respiratory distress. The important dates in this study are the years 1970s or the early 1980s. The events happening in this document are that researchers reviewed 4 studies to determine the effectiveness of early CPAP therapy compared to delayed CPAP in lowering mortality rate and reducing the need for intermittent positive‐pressure ventilation in infants with respiratory distress. The result of these events is that the evidence is outdated. It is also unclear whether early CPAP therapy compared to delayed CPAP had an effect in lowering mortality rate and use of intermittent positive‐pressure ventilation. The take-home message based on the results is that there is lack of evidence to support that whether early application of CPAP has clinical benefit in the treatment of respiratory distress, or whether it is associated with any adverse effects. Further research is required.” “Lay Summary: Early versus delayed initiation of continuous positive airway pressure (CPAP) for respiratory distress in preterm infants Review question We wanted to find out whether for preterm infants in respiratory distress, applying continuous positive airway pressure (CPAP) early would result in increased benefit and less harm than if it were applied later. Background Preterm babies often lack surfactant, a detergent‐like substance produced by the lung. Lack of surfactant causes their lungs to fail to expand properly at birth and results in the need for greater effort in breathing. If left untreated, the breathing difficulty progressively worsens and may lead to lung damage. CPAP improves expansion of the lung, making it easier for the baby to breathe, and might reduce the need for intermittent positive‐pressure ventilation (IPPV), a form of respiratory support that carries greater risks, including the risk of developing a type of lung damage called bronchopulmonary dysplasia (BDP). CPAP might also reduce the risk of the baby dying from respiratory distress. CPAP is applied through a face mask, a nasal mask, or prongs into the nostrils. Study characteristics: The search is up‐to‐date as of June 2020. We found four small studies including a total of 119 babies. All four studies were performed in the 1970s or early 1980s, when the use of antenatal steroids (given to the mother to help a preterm baby's lung to become more mature) was uncommon. Key results From these four small studies, we are very uncertain whether early CPAP provides any benefit or whether it causes any harm. Certainty of evidence All four included studies had weaknesses in the way they were conducted, and all were very small. In addition, because they were old studies, study results might not apply to the current care of preterm infants. Therefore, we assessed the certainty of evidence as very low.”}" [Examples from the Cochrane Database of Systematic Reviews^1^]

Generated Education: “Act as an educator and write a lay summary using the study abstract. The goal is to simplify the abstract for non-experts and make the study easily accessible and understandable for the broader public. Generate a title for the summary. Comment on the quality of evidence. Consider what a good lay summary should have. Apply these considerations into generating a lay summary.”

GPT-Plugin tool (Prompt Perfect): “Create a simplified lay summary based on a study abstract, aimed at non-experts. The summary should be easy to understand, capturing the main findings, purpose, and significance of the study, while avoiding technical jargon. Focus on explaining what the study was about, why it was important, and what the results mean for everyday life. Keep it clear and concise for a broad audience."

The prompts combined with the study abstracts were sent to the LLMs using their API key on Google Colab. Additionally, the hyperparameters - temperature and Top_p – of the LLMs were set to 0.25, 0.5, 0.75 or 1.0 increments alternatively. This process was repeated three times per abstract, and LLM. The three outputs were quantitatively measured using cosine similarity against the expert-drafted summaries from the dataset. Descriptive statistical analysis was performed on the cosine similarity scores, and the highest median cosine similarity score was calculated.

**Results**

**Table A: The prompt style per Large Language Model that generated lay summaries with the highest median cosine similarity score.**

| Models | Prompt Style | Highest Median Cosine Similarity Score |
| --- | --- | --- |
| Gemini- 1.5- Flash | Generated Knowledge | 0.6225 |
| Gemini – 1.5 – Pro | Generated Knowledge | 0.6281 |
| GPT – 4o - mini | Generated Knowledge | 0.6688 |
| GPT – 4 | Few-Shot | 0.6852 |
| Llama 3.1 | Zero-Shot | 0.6381 |

Table A contains the highest median similarity scores of lay summaries generated per LLM. GPT-4 with Few-Shot yielded the highest score (0.6850), followed by GPT-4o mini (0.6680).

In contrast, Gemini 1.5 - Flash had the lowest median cosine similarity (0.6225), while Gemini – 1.5 – Pro had the second lowest score (0.6281). This indicates that GPT 4 family models under few-shot and generated knowledge produced lay summaries that were the most semantically similar to the expert-generated lay summaries.

**Table B: The hyperparameter combinations (Temperature and Top_p) per Large Language Model that generated lay summaries with the highest median cosine similarity score.^2^**

| Models | Hyperparameter Combinations  (Temperature value, Top_p value) | Highest Median Cosine Similarity Score |
| --- | --- | --- |
| Gemini- 1.5- Flash | (0.75, 1.0) | 0.56 |
| Gemini – 1.5 – Pro | (0.75, 0.75) | 0.55 |
| GPT – 4o - mini | (1.0, 0.75) | 0.62 |
| GPT – 4 | (0.5, 1.0) | 0.64 |
| Llama 3.1 | (0.5, 1.0) | 0.57 |

Table B shows that GPT 4 and GPT-4o mini had the highest median cosine similarity score compared to other LLMs across all hyperparameter combinations. Results of the hyperparameter combinations are further discussed in the supplementary files of a previously published paper.^2^

**Conclusion**

The best performing prompt and hyperparameter combinations per LLM in this pilot study were used for the main study.

**References**

1. Guo Y, Qiu W, Wang Y, Cohen T. Automated Lay Language Summarization of Biomedical Scientific Reviews. *35th AAAI Conference on Artificial Intelligence, AAAI 2021*. 2020;1:160-168. doi:10.1609/aaai.v35i1.16089
2. Tasneem N, van der Pol CB, Zahoor A, Juggath N, McGowan K, Lokker C, Saha A. From radiology findings to artificial intelligence (AI) powered impressions: a retrospective study on the comparative performance of recent large language models. *Intelligent Medicine*. 2025 Dec 1. doi:10.1016/j.imed.2025.11.003.
