# Supplementary material for: Evaluating large language models for lay summaries of radiology reports using tailored prompting strategies and mixed-method assessment
Source: PLOS Digit Health. 2026 Sep 17;5(9):e0001672. doi: 10.1371/journal.pdig.0001672 (PMC13585270; doi:10.1371/journal.pdig.0001672)
Supplement: Supplementary file 3 [file pdig.0001672.s003.docx]

## **S3 Text. Expert and LRM evaluation questionnaire**

**Expert Evaluation for Generated Lay Summary^2^**

1. "The simplified version does not contain any inaccurate or misleading information"
   - 1 = Strongly Disagree
   - 2 = Disagree
   - 3 = Neither Agree or Disagree
   - 4 = Agree
   - 5 = Strongly Agree
2. "The simplified version includes all relevant/actionable information present in the original impression"
   - 1 = Strongly Disagree
   - 2 = Disagree
   - 3 = Neither Agree or Disagree
   - 4 = Agree
   - 5 = Strongly Agree
3. "The simplified version offers beneficial supplementary information not found in the original impression"
   - 1 = Strongly Disagree
   - 2 = Disagree
   - 3 = Neither Agree or Disagree
   - 4 = Agree
   - 5 = Strongly Agree
4. The simplified version is:
   - Accurate and Concise
   - Inaccurate but Concise
   - Accurate but Lengthy
   - Inaccurate and Lengthy
5. “The simplified version of the radiology report contains extraneous information.”
   - 1 = Strongly Disagree
   - 2 = Disagree
   - 3 = Neither Agree or Disagree
   - 4 = Agree
   - 5 = Strongly Agree

6. "I feel comfortable giving the simplified output to patients without any supervision."
   - 1 = Strongly Disagree
   - 2 = Disagree
   - 3 = Neither Agree or Disagree
   - 4 = Agree
   - 5 = Strongly Agree
1. Which summary completely captures more important information?
   - Lay Summary A
   - Lay Summary B
   - Lay Summary C
   - Lay Summary D
   - Lay Summary E
2. Which summary includes less false information?
   - Lay Summary A
   - Lay Summary B
   - Lay Summary C
   - Lay Summary D
   - Lay Summary E
3. Which summary contains less non-important information?
   - Lay Summary A
   - Lay Summary B
   - Lay Summary C
   - Lay Summary D
   - Lay Summary E

**Prompt for Large Reasoning Model:**

“" You are a board-certified radiologist with 10 years and more experience and you are evaluating a generated lay summary of a radiology report based on expert criteria. Please respond in the following JSON format:

{ ""misleading"": ""The simplified version does not contain any inaccurate or misleading information. Choose one: Strongly Agree | Agree | Neither Agree or Disagree | Disagree | Strongly Disagree"",

""actionable_information"": ""The simplified version includes all relevant/actionable information present in the original impression. Choose one: Strongly Agree | Agree | Neither Agree or Disagree | Disagree | Strongly Disagree"",

""beneficial_info"": ""The simplified version offers beneficial supplementary information not found in the original impression. Choose one: Strongly Agree | Agree | Neither Agree or Disagree | Disagree | Strongly Disagree"",

""conciseness"": ""The simplified version is (Choose one): Accurate and Concise | Inaccurate but Concise | Accurate but Lengthy | Inaccurate and Lengthy"",

""extraneous_info"": ""The simplified version of the radiology report contains extraneous information. Choose one: Strongly Agree | Agree | Neither Agree or Disagree | Disagree | Strongly Disagree"",

""without_supervision"": ""I feel comfortable giving the simplified version to patients without any supervision. Choose one: Strongly Agree | Agree | Neither Agree or Disagree | Disagree | Strongly Disagree"",}

This is the original report (for context): {original report}

--- BEGIN SUMMARY --- {summary} --- END SUMMARY --- “””

“You are a board-certified radiologist with 10 years and more experience and you are evaluating 5 AI generated lay summaries of a radiology report.

{Lay Summaries}

Compare the 5 lay summaries using the expert criteria provided below and please respond in the following JSON format:

{ ""More_Important"": ""Which summary completely captures more important information? Choose one of the options: Lay Summary A | Lay Summary B | Lay Summary C | Lay Summary D | Lay Summary E"",

""Less_False"": ""Which summary includes less false information? Choose one of the options: Lay Summary A | Lay Summary B | Lay Summary C | Lay Summary D | Lay Summary E"",

""Less_NonImportant"": ""Which summary contains less non-important information? Choose one of the options: Lay Summary A | Lay Summary B | Lay Summary C | Lay Summary D | Lay Summary E"", }

The original report that was used to generate all 5 summaries has been provided for additional context: {Original Report}
