# Supplementary material for: Evaluating large language models for lay summaries of radiology reports using tailored prompting strategies and mixed-method assessment
Source: PLOS Digit Health. 2026 Sep 17;5(9):e0001672. doi: 10.1371/journal.pdig.0001672 (PMC13585270; doi:10.1371/journal.pdig.0001672)
Supplement: Supplementary file 4 [file pdig.0001672.s004.docx]

## **S4 Text. Inter-rater reliability metric agreement matrices**

**Figure 1: Agreement matrices showing count comparisons between Human Raters 1 and 2 for each Large Language Model and Likert criteria.**

From Figure 1, Human rater 1 and 2 largely agreed in all criteria for Gemini 1.5 – Flash and Pro. For Beneficial Information and Supervision criteria in GPT-4o mini, GPT-4 and Llama 3.1, both raters chose “Neither Agree or Disagree” (shown as “Neutral”) option more frequently. Llama 3.1 had a high count of disagreements with the largest disagreement occurring for the Supervision criteria.
