# Supplementary material for: Evaluating large language models for lay summaries of radiology reports using tailored prompting strategies and mixed-method assessment
Source: PLOS Digit Health. 2026 Sep 17;5(9):e0001672. doi: 10.1371/journal.pdig.0001672 (PMC13585270; doi:10.1371/journal.pdig.0001672)
Supplement: Supplementary file 5 [file pdig.0001672.s005.docx]

## **S5 Text. Percentage Agreement Rankings**

LLM ranking using percentage agreement

**Table A: Percentage Agreement and Ranking of Likert Statements between human experts.**

|  | **Percentage Agreement (Ranking)** | | | | |
| --- | --- | --- | --- | --- | --- |
| Likert Statements | Gemini 1.5 - Flash | Gemini 1.5 - Pro | GPT-4o mini | GPT-4 | Llama 3.1 |
| The simplified version does not contain any inaccurate or misleading information.  (↑) | 84% (1) | 80% (2) | 67% (3) | 66% (4) | 16% (5) |
| 'The simplified version includes all relevant/actionable information present in the original impression. (↑) | 81% (1) | 78% (2) | 62% (4) | 65% (3) | 21% (5) |
| The simplified version offers beneficial supplementary information not found in the original impression. (↑) | 57% (1) | 45% (2) | 43% (3) | 31% (4) | 9% (5) |
| The simplified version of the radiology report contains extraneous information. (↓) | 0% (1) | 0% (1) | 1% (2) | 0% (1) | 27% (3) |
| I feel comfortable giving the simplified version to patients without any supervision. (↑) | 70% (2) | 71% (1) | 29% (3) | 18% (4) | 1% (5) |
| **Average of Ranking** | 1 | 1.8 | 3 | 3.2 | 4.6 |

Note: This table presents the percentage of responses that were Agree or Strongly Agree for each of the five evaluation criteria. Rankings were assigned based on the percentage agreement, with higher agreement (↑) corresponding to a higher ranking. For Extraneous Information, the ranking is reversed, such that a lower percentage agreement (↓) corresponds to a higher ranking, reflecting less unnecessary information.

**Table B: Percentage Agreement and Ranking of Likert Statements among the human experts and Gemini 2.5 – Pro**

|  | **Percentage Agreement (Ranking)** | | | | |
| --- | --- | --- | --- | --- | --- |
| Likert Statements | Gemini 1.5 - Flash | Gemini 1.5 - Pro | GPT-4o mini | GPT-4 | Llama 3.1 |
| The simplified version does not contain any inaccurate or misleading information. (↑) | 82% (1) | 75% (2) | 58% (3) | 57% (4) | 8% (5) |
| 'The simplified version includes all relevant/actionable information present in the original impression. (↑) | 76% (1) | 69% (2) | 56% (4) | 57% (3) | 10% (5) |
| The simplified version offers beneficial supplementary information not found in the original impression. (↑) | 57% (1) | 45% (2) | 43% (3) | 30% (4) | 9% (5) |
| The simplified version of the radiology report contains extraneous information. (↓) | 0% (1) | 0% (1) | 0% (1) | 0% (1) | 21% (2) |
| I feel comfortable giving the simplified version to patients without any supervision. (↑) | 60% (2) | 61% (1) | 24% (3) | 13% (4) | 1% (5) |
| **Average of Ranking** | 1.2 | 1.6 | 3.2 | 3.2 | 4.4 |

Note: This table presents the percentage of responses that were Agree or Strongly Agree for each of the five evaluation criteria. Rankings were assigned based on the percentage agreement, with higher agreement (↑) corresponding to a higher ranking. For Extraneous Information, the ranking is reversed, such that a lower percentage agreement (↓) corresponds to a higher ranking, reflecting less unnecessary information.

**Table C: Percentage Agreement and Ranking of Likert Statements among the human experts and GPT-oss-120b**

|  | **Percentage Agreement (Ranking)** | | | | |
| --- | --- | --- | --- | --- | --- |
| Likert Statements | Gemini 1.5 - Flash | Gemini 1.5 - Pro | GPT-4o mini | GPT-4 | Llama 3.1 |
| The simplified version does not contain any inaccurate or misleading information. (↑) | 69% (2) | 71% (1) | 59% (3) | 54% (4) | 4% (5) |
| 'The simplified version includes all relevant/actionable information present in the original impression. (↑) | 67% (1) | 61% (2) | 46% (4) | 55% (3) | 8% (5) |
| The simplified version offers beneficial supplementary information not found in the original impression. (↑) | 27% (1) | 18% (4) | 20% (3) | 23% (2) | 4% (5) |
| The simplified version of the radiology report contains extraneous information. (↓) | 0% (1) | 0% (1) | 0% (1) | 0% (1) | 24% (2) |
| I feel comfortable giving the simplified version to patients without any supervision. (↑) | 53% (2) | 54% (1) | 21% (3) | 15% (4) | 0% (5) |
| **Average of Ranking** | 1.4 | 1.8 | 2.8 | 2.8 | 4.4 |

Note: This table presents the percentage of responses that were Agree or Strongly Agree for each of the five evaluation criteria. Rankings were assigned based on the percentage agreement, with higher agreement (↑) corresponding to a higher ranking. For Extraneous Information, the ranking is reversed, such that a lower percentage agreement (↓) corresponds to a higher ranking, reflecting less unnecessary information.
