# Supplementary material for: Evaluating large language models for lay summaries of radiology reports using tailored prompting strategies and mixed-method assessment
Source: PLOS Digit Health. 2026 Sep 17;5(9):e0001672. doi: 10.1371/journal.pdig.0001672 (PMC13585270; doi:10.1371/journal.pdig.0001672)
Supplement: Supplementary file 6 [file pdig.0001672.s006.docx]

## **S6 Text. Statistical significance between length of findings section and lay summary quality per rater**

For human rater 1, the relationship between the length of the findings section and lay summary quality varied across models and evaluation criteria. Longer findings were significantly associated with increased extraneous information for GPT-4o mini (ρ = 0.259, *P* = 0.009) and GPT-4 (ρ = 0.343, *P* < 0.001), as well as a weaker but significant association for Llama 3.1 (ρ = 0.243, P = 0.015). However, inclusion of relevant or actionable information demonstrated a negative correlation with findings length for GPT-4o mini (ρ = –0.213, P = 0.033). No significant associations were observed for inaccurate or misleading content, beneficial supplementary information, or rater comfort in providing summaries to patients without supervision across most models.

For human rater 2, longer findings were consistently associated with increased extraneous information across multiple models, including Gemini 1.5 - Flash (ρ = 0.288, P = 0.004), Gemini 1.5 - Pro (ρ = 0.287, P = 0.004), GPT-4o mini (ρ = 0.304, P = 0.002), and Llama 3.1 (ρ = 0.406, P < 0.001). In contrast, longer findings were negatively correlated with the inclusion of relevant or actionable information for Gemini 1.5 - Pro (ρ = –0.382, P < 0.001). Similarly, ratings for inclusion of misleading information showed significant negative associations for Gemini 1.5 - Pro (ρ = –0.395, P < 0.001) and GPT-4 (ρ = –0.256, P = 0.010), as did rater comfort in providing unsupervised summaries for Gemini 1.5 - Pro (ρ = –0.416, P < 0.001) and GPT-4 (ρ = –0.250, P = 0.012). Across all models, beneficial supplementary information demonstrated negative correlation with findings length, most notably for Gemini 1.5 - Pro (ρ = –0.355, P < 0.001) and GPT-4 (ρ = –0.262, P = 0.009).

For the LRM evaluator, longer findings were significantly associated with decreased ratings for inclusion of relevant/actionable information across multiple models, including Gemini 1.5 - Flash (ρ = –0.208, P = 0.038), GPT-4o mini (ρ = –0.340, P < 0.001), GPT-4 (ρ = –0.522, P < 0.001), and Llama 3.1 (ρ = –0.265, P = 0.008). Inclusion of inaccurate/misleading information also showed negative correlations with findings length for GPT-4o mini (ρ = –0.213, P = 0.034) and GPT-4 (ρ = –0.290, P = 0.003). Similarly, rater comfort in providing summaries without supervision decreased with longer findings for Gemini 1.5 - Flash (ρ = –0.253, P = 0.011), Gemini 1.5 - Pro (ρ = –0.326, P = 0.001), GPT-4o mini (ρ = –0.354, P < 0.001), and GPT-4 (ρ = –0.562, P < 0.001). In contrast, longer findings were positively associated with extraneous information for GPT-4 (ρ = 0.553, P < 0.001), while correlations for other models were weaker or nonsignificant. Beneficial supplementary information showed mostly weak and nonsignificant negative associations with findings length, with the exception of GPT-4 (ρ = –0.201, P = 0.045).
